# Platinum Nanoparticles Decorated on NaNbO_3_ and LiNbO_3_ as Catalysts for Enhanced Hydrogen Generation via NaBH_4_ Hydrolysis

**DOI:** 10.1021/acsomega.6c02694

**Published:** 2026-06-13

**Authors:** Iterlandes Machado Junior, Matheus Araújo Pereira, Gabriel Henrique Sperandio, Kleryton Luiz Alves de Oliveira, Antonio Machado Netto, Fabrício Vieira de Andrade, Renata Pereira Lopes Moreira

**Affiliations:** † Department of Chemistry, 28120 Universidade Federal de Viçosa (UFV), Av. Peter Henry Rolfs, s/n, Campus Universitário, 36.570-000 Viçosa, Minas Gerais, Brazil; ‡ Universidade Federal de Itajubá (UNIFEI), Campus Itabira, Rua Irmã Ivone Drumond, 200, Distrito Industrial II, 35903087 Itabira, Minas Gerais, Brazil

## Abstract

Alkali-metal niobates MNbO_3_ (M = Na, Li) decorated with platinum (Pt) nanoparticles (NPs) are innovatively utilized as catalytic supports for hydrogen (H_2_) evolution from sodium borohydride (NaBH_4_). Notably, a direct comparison between sodium niobate (NaNbO_3_) and lithium niobate (LiNbO_3_) in their efficiency for H_2_ evolution from NaBH_4_ was conducted for the first time, highlighting the distinct catalytic performance of these materials. The synthesis was confirmed by X-ray diffraction (XRD), scanning electron microscopy (SEM), and high-resolution transmission electron microscopy (HRTEM). The deposited Pt nanoparticles remain below 10 nm. In this study, different transition-metal nanoparticles (M = Co, Ni, Pd, Pt) supported on NaNbO_3_ and LiNbO_3_ were evaluated as catalysts. The Pt NPs/NaNbO_3_ and Pt NPs/LiNbO_3_ catalysts demonstrated optimized performance, producing H_2_ at similar rates of 2044.9 and 2303.7 mL min^–1^ g_cat_^–1^ at 293.15 K. Arrhenius plots indicate activation energies (*E*_a_) of 35.54 ± 1.32 kJ mol^–1^ for Pt NPs/NaNbO_3_ and 35.04 ± 1.32 kJ·mol^–1^ for Pt NPs/LiNbO_3_. Additionally, the materials exhibited consistent stability, with an efficiency loss of less than 20% after the 10th cycle of use. These results underscore the potential of these catalysts for H_2_ generation applications.

## Graphical abstract

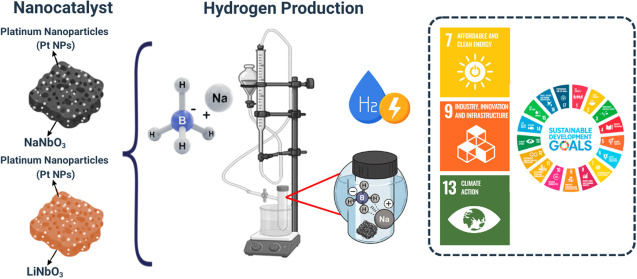

## 1 Introduction

The increasing frequency of severe climate events and widespread reliance on fossil fuels underscore the importance of transitioning gradually to more sustainable energy sources. Consequently, the adoption of hydrogen as an energy source emerges as a compelling alternative, significantly mitigating greenhouse gas emissions.^1,2^ Due to hydrogen’s high flammability and low volumetric energy density, substantial challenges are encountered in ensuring safe and efficient storage, transport, and release.^3,4^ For this purpose, inorganic hydrides come into play as a viable hydrogen storage technology, helping to overcome challenges related to its handling and transportation.^5^ The main inorganic hydrides employed include NH_3_BH_3_,^6–8^ NaBH_4_,^9–15^ B_2_(OH)_4_,^16^ and LiH,^17^ M­(AlH_4_) wherein M = Na, Li, and K,^18^ among others. Amine–borane derivatives such as ethylenediamine bisborane (EDAB) have also been investigated as hydrogen storage materials due to their relatively high gravimetric hydrogen content (∼16.1 wt %) and improved properties compared to conventional systems.^19^ EDAB has been reported as an alternative to ammonia borane, with faster hydrogen release kinetics and lower reaction enthalpy.^20^

NaBH_4_ is one of the most studied inorganic hydrides due to its low toxicity, high stability in alkaline solution, nonflammability, and theoretical hydrogen storage capacity of 10.8 wt %.^21^ The NaBH_4_ hydrolysis reaction is shown in eq 1

1 $${\mathrm{NaBH}}_{4\left(\mathrm{aq}\right)}+4H_{2}O_{\left(l\right)}\to {B{\left(\mathrm{OH}\right)}_{4\left(\mathrm{aq}\right)}}^{-}+4H_{2\left(g\right)}+210\;\mathrm{kJ}$$

The reaction of sodium borohydride can proceed without a catalyst. However, self-hydrolysis is slow at room temperature.^22^ To overcome this limitation, efficient hydrogen production from NaBH_4_ can be achieved using an appropriate catalyst, which also enables greater control over the reaction.^23^ Nonnoble-metal catalysts such as Co^14,15,22,24–27^ and Ni,^15,28–33^ offer economic advantages, but their catalytic performance is generally lower compared to noble-metal catalysts like Ru^8,34–36^ and Pt.^36–40^ In this context, nanoscale engineering has emerged as an effective strategy to enhance catalytic performance while simultaneously reducing the required metal loading. The physicochemical properties of nanoparticles play a fundamental role in determining their performance and applicability in advanced technological systems.^41^

The incorporation of the support is crucial in this process, preventing nanoparticle agglomeration. These supports not only hinder the agglomeration of active particles^36^ but also facilitate their separation from the solution.^42^ In this regard, advanced surface engineering and bonding strategies applied to tailored support frameworks have proven vital for stabilizing active phases and inhibiting thermal or chemical aggregation.^43^ Furthermore, the architectural design of the support–metal interface is critical, as specific structure–function relationships can significantly modulate the physicochemical stability of the resulting nanoparticles.^44^ In this regard, tailoring the electronic structure and orbital configurations across adjacent domains plays a fundamental role in decoupling and shifting localized reaction barriers.^45^ Similarly, the architectural design of composite systems highlights the importance of support configurations in accelerating interfacial catalytic reactions.^46^ Efficient catalytic supports must prioritize high surface accessibility and structural integrity, as precisely designed crystalline frameworks optimize charge-carrier transport and ensure stability in harsh reaction media.^47^

Recent studies have shown that the stabilization of metallic nanoparticles (NPs), such as Pt, Ni, Pd, and Au, in niobic acid results in a highly efficient microheterogeneous catalytic system for the hydrolysis of NaBH_4_ under mild conditions.^11^ Niobic acid, a polymorphic form of Nb_2_O_5_, was used for the first time in this reaction, highlighting its Brønsted acidity’s key role in catalytic activity through synergistic interactions with the metallic NPs.^11^ Multicomponent nanomaterials have shown great potential for expanding functional versatility due to synergistic effects between their constituent elements.^48^ Additionally, cobalt-modified niobium oxide-based catalysts exhibited significant performance in hydrogen generation via NaBH_4_ hydrolysis, highlighting the role of niobium in stabilizing the active catalytic phases and enhancing catalytic activity.^49^ The synergy between the niobium-based support and metallic NPs in NaBH_4_ hydrolysis occurs through three main mechanisms: (i) enhanced dispersion and stability of the nanoparticles, preventing agglomeration and maximizing the active surface area; (ii) electronic interactions between the support and the nanoparticles, which modify their catalytic properties, reduce the activation energy of the reaction, and are aligned with strategies that utilize localized spin polarization and electronic asymmetries to effectively reduce reaction barriers;^50^ and (iii) the acidity of niobium, which promotes water adsorption and activation, a crucial step in NaBH_4_ hydrolysis.^49^ Thus, the presence of niobium not only stabilizes the metallic NPs but also enhances their catalytic activity, leading to a more efficient hydrogen release.

Alkali-metal niobates exhibit multiple distinct electrical and mechanical properties. NaNbO_3_ is commonly used in devices for electric charge storage,^51^ ferroelectric memories,^52,53^ humidity sensors,^54^ pressure transducers,^55,56^ and electromechanical systems.^55,56^ On the other hand, LiNbO_3_ possesses remarkable electromechanical properties, capable of significantly altering optical properties in response to an electric field.^57^ These characteristics make LiNbO_3_ a valuable material in optical and electrooptical applications. Due to their chemical stability, ease of preparation, and being semiconductors with a wide bandgap, generating high-energy electron/hole pairs, they are suitable for the degradation of organic pollutants.^57–59^ To date, there is a significant gap in research related to the hydrolysis of sodium borohydride using catalysts deposited on alkali-metal niobates.

Several works involving platinum nanoparticles on different supports are described in the literature. Altaf and co-workers employed a titanium dioxide support modified with ruthenium and platinum for the hydrolysis of NaBH_4_.^36^ According to the authors, the Pt/TiO_2_ catalyst exhibited a hydrogen generation rate (HGR) of 140 mL·min^–1^·g_cat_^–1^ (at 25 °C) and demonstrated reusability over six cycles. Dai et al. used Pt/Al_2_O_3_ over cordierite monolith (2MgO·2Al_2_O_3_·5SiO_2_) as catalyst for the hydrolysis of NaBH_4_ in a flow reactor.^42^ According to the authors, the catalyst exhibited excellent stability against metal aggregation and leaching. Huff et al. synthesized platinum nanoparticles stabilized with β-cyclodextrin.^37^ The system presented an activation energy of 39.2 kJ mol^–1^ and HGR of 0.816 mL min^–1^ mL_cat_^–1^ at 22 °C (295 K) under pH 7. Biehler and co-workers synthesized platinum nanoparticles supported on fused nanosized carbon spheres.^40^ This material was utilized in the hydrolysis of NaBH_4_, demonstrating an activation energy of 53.0 kJ mol^–1^ and HGR of 0.0438 mL min^–1^ mg_cat_^–1^ at pH 6 and 25 °C. Uzundurukan and Devrim synthesized a platinum catalyst supported on multiwalled carbon nanotubes, which exhibited an activation energy of 27.0 kJ mol^–1^.^39^

As mentioned previously, the search for efficient and stable catalysts for hydrogen production remains a key challenge in the development of sustainable energy technology. In this context, Pt NPs supported on alkali-metal niobates (Pt NPs/NaNbO_3_ and Pt NPs/LiNbO_3_) have emerged as promising candidates due to their exceptional catalytic activity in the alkaline hydrolysis of NaBH_4_. It is also worth highlighting that niobium is a strategic and predominantly Brazilian resource, which adds significant economic and geopolitical value to niobate-based catalytic systems in the context of sustainable hydrogen technology. In particular, the synergistic interaction between Pt NPs and the niobate support, associated with intrinsic acidity and electronic properties of niobium-based materials, can enhance metal dispersion, promote water activation, and improve the catalytic efficiency in NaBH_4_ hydrolysis. To the best of our knowledge, this is the first study to directly compare the catalytic performance of platinum nanoparticles supported on NaNbO_3_ and LiNbO_3_ for hydrogen generation via NaBH_4_ hydrolysis, providing valuable insights into the influence of niobate support composition on catalytic efficiency and reaction kinetics.

## 2 Results and Discussion

### 2.1 Catalyst Characterization

The catalyst was characterized since structure–property relationships are essential for understanding the physicochemical behavior and potential applicability of functional materials.^60^ In this regard, comprehensive structural validation through advanced spectroscopic and analytical techniques is fundamental to establishing reliable correlations between a material’s architecture and its functional performance.^61^ Figure 1 displays the diffractograms of the synthesized niobates. The diffraction peaks demonstrate that the hydrolysis mechanism of the synthesized LiNbO_3_ (Figure 1a) aligns perfectly with the rhombohedral lithium niobate reference pattern (JCPDS No. 01-085-2456), confirming the successful synthesis of this niobate via the Pechini method.

**Figure 1:**
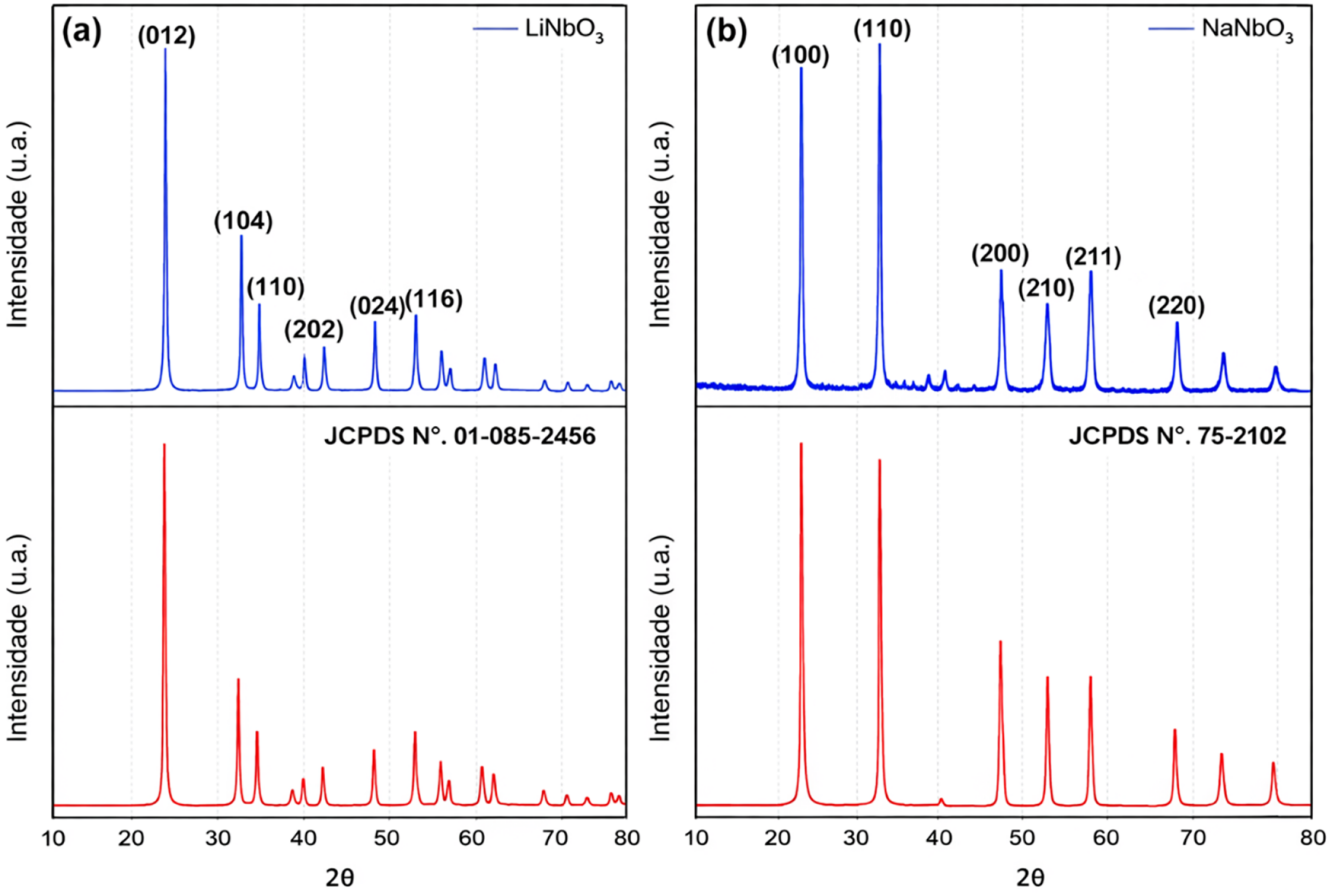
Diffractograms obtained for (a) lithium niobate and (b) sodium niobate synthesized samples.

Similarly, Figure 1b presents the diffraction peaks of the synthesized NaNbO_3_. The diffractogram aligns well with the cubic sodium niobate reference pattern (JCPDS No. 75-2102), confirming the successful synthesis of NaNbO_3_ via the Pechini method. Moreover, the absence of peaks corresponding to secondary phases suggests that the obtained materials are of high purity. Furthermore, the crystallite sizes of the materials were determined via Scherrer analysis of the XRD data, yielding values of 35.13 nm for NaNbO_3_ and 33.70 nm for LiNbO_3_. Skjærvø et al.^62^ reported similar results for NaNbO_3_ synthesis, obtaining crystallite sizes ranging from 35 to 50 nm.

Figure S1 shows SEM images of the synthesized materials, revealing a rough morphology for both materials. Figure 2(a–c) and (d–f) shows the HRTEM images of LiNbO_3_ and Pt NPs/LiNbO_3_, respectively. High-resolution transmission electron microscopy was employed to estimate the particle sizes and to gather detailed information about the interplanar spacings of the synthesized materials. Figure 2d,e shows the Pt NPs formed on the surface of the LiNbO_3_ support. Figure 2a displays the formed LiNbO_3_ support particles. Figure 2g presents the corresponding size distribution histogram, showing an average size of 32.96 ± 2.60 nm. This result is in good agreement with the crystallite size obtained from Scherrer analysis of the XRD data (33.70 nm). Furthermore, Figure 2h presents the particle size distribution histogram, showing an average size of 6.57 ± 1.22 nm.

**Figure 2:**
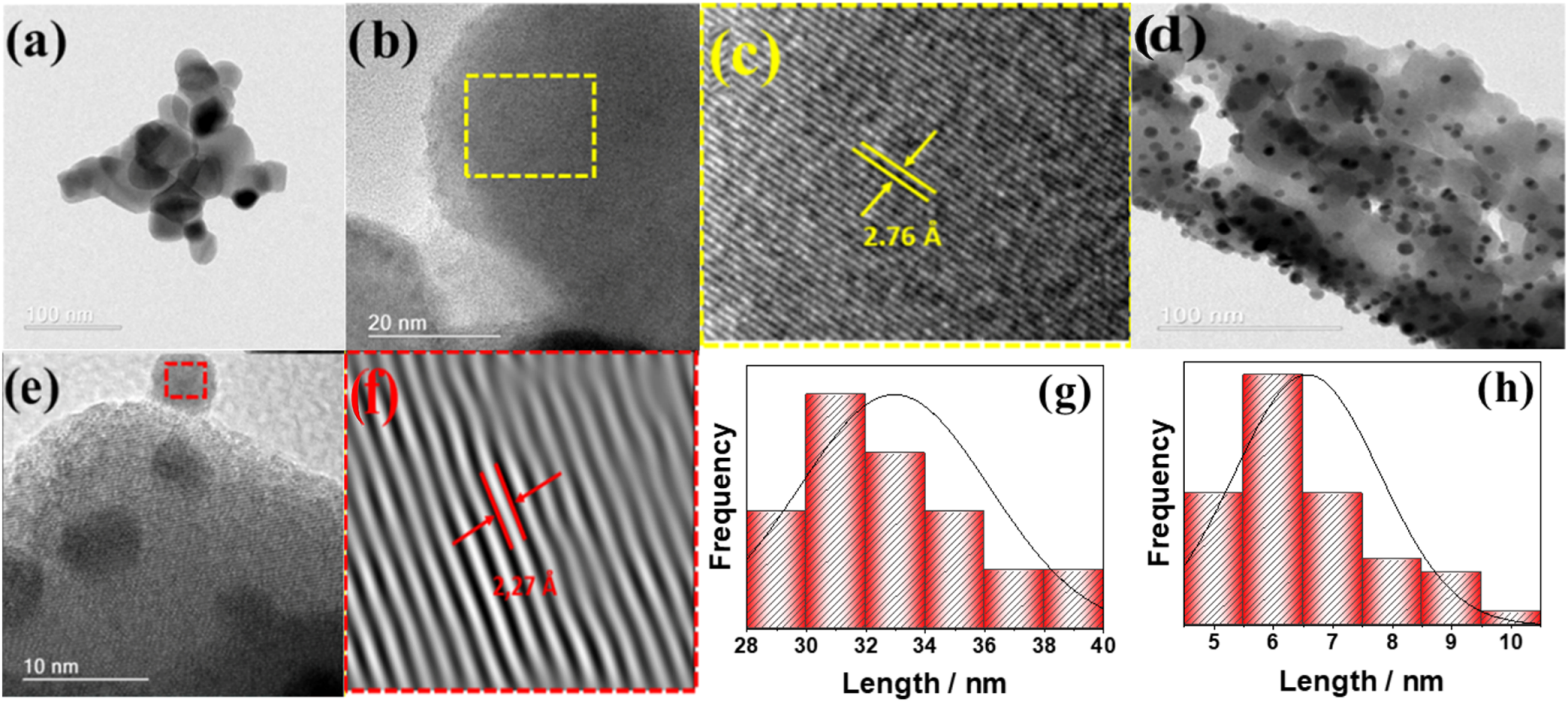
HRTEM images of synthesized LiNbO_3_ (a–c), Pt NPs-LiNbO_3_ (d–f), and histogram of the particle size distribution of the support (g) and Pt (h).

Additionally, fast Fourier transform (FFT) analysis was utilized to enhance the visualization of interplanar distances in the HRTEM images. The regions selected for analysis are marked by a yellow square for LiNbO_3_ (Figure 2b) and a red square for Pt NPs/LiNbO_3_ (Figure 2f). The points on the plot represent the periodicity of the crystal planes observed in the HRTEM images. By isolating these points from noise and applying the inverse Fourier transform (inverse FFT), a reconstructed microscopic image was obtained. This process enhanced the clarity of the crystallographic planes, enabling precise measurement of interplanar distances from the HRTEM images. The images obtained from this process are shown in Figure 2c,f. The HRTEM images confirm the high crystallinity of the nanoparticles. The lattice fringe with a spacing of 2.76 Å (Figure 2c) corresponds to the interplanar distance of the (104) planes of LiNbO_3_ (JCPDS No. 01-085-2456), associated with the second most intense peak in the sample’s diffractogram (Figure 1a). Similarly, Figure 2f shows the lattice fringe for a platinum nanoparticle deposited on the surface of LiNbO_3_. The measured interplanar distance of 2.27 Å corresponds to the (111) planes of metallic platinum (JCPDS No. 1-1190). These results demonstrate the efficiency of the deposition process for metallic nanoparticles on the LiNbO_3_ surface. The structural integrity and phase stability of such metallic nanoparticles are paramount, as nanoscale validation is essential to confirm the formation of stable interfaces.^63^

Similar to the treatment applied to the HRTEM images of LiNbO_3_, Figure 3(a,b) and (d,e) presents the HRTEM images of NaNbO_3_ and Pt NPs/NaNbO_3_, respectively. Likewise, Figure 3d,e shows the Pt NPs formed on the surface of the NaNbO_3_ support, and Figure 3h presents the particle size distribution histogram, showing an average size of 6.51 ± 1.75 nm, consistent with observations for the previous material. Furthermore, Figure 3a,b displays the formed NaNbO_3_ support particles, while Figure 3g presents the corresponding size distribution histogram, showing an average size of 35.45 ± 1.98 nm. This result is in good agreement with the crystallite size obtained from Scherrer analysis of the XRD data (35.13 nm). This result highlights the reproducibility of the synthesis method across different materials. Fast Fourier transform (FFT) was again employed to enhance the visualization of interplanar distances in the HRTEM images.

**Figure 3:**
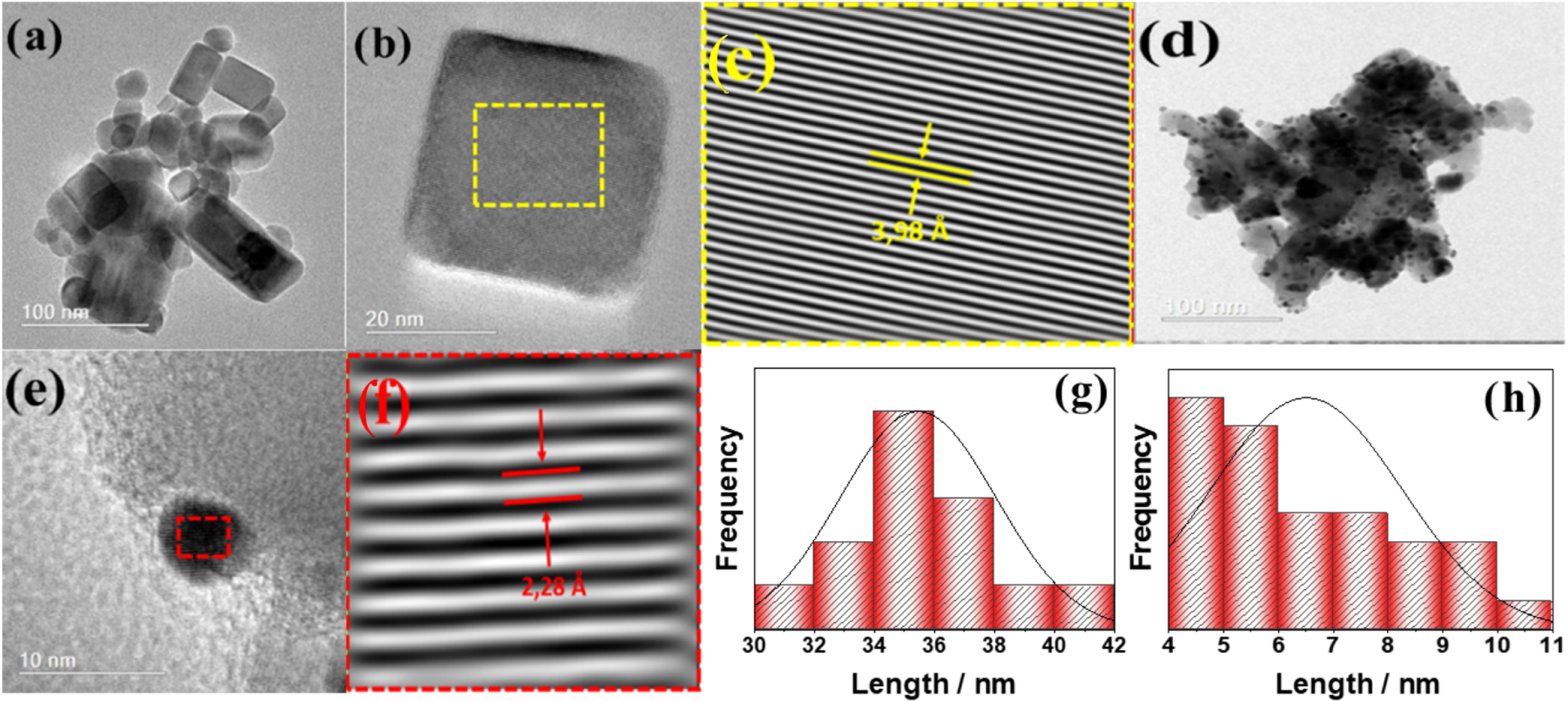
HRTEM images of synthesized NaNbO_3_ (a–c), Pt NPs-NaNbO_3_ (d–f), and histogram of the particle size distribution of the support (g) and Pt (h).

The regions analyzed are marked by a yellow square for NaNbO_3_ (Figure 3b) and a red square for Pt NPs-NaNbO_3_ (Figure 3f), following the same approach as for the earlier materials. The processed HRTEM images are shown in Figure 3c,f. The TEM images confirm the high crystallinity of nanoparticles. The lattice fringe with a spacing of 3.98 Å, observed in Figure 3c, corresponds to the interplanar distance of the (100) planes of cubic NaNbO_3_ (JCPDS No. 75-2102), associated with the second most intense peak in the diffractogram (Figure 1b). Similarly, the lattice fringe observed in a platinum nanoparticle deposited on the surface of NaNbO_3_ (Figure 3f) shows an interplanar distance of 2.28 Å, which can be attributed to the (111) planes of metallic platinum (JCPDS No. 1-1190).

Scanning electron microscopy (SEM) images (Figure 4) revealed a more aggregated and rough morphology for NaNbO_3_, whereas LiNbO_3_ exhibited a more compact and lamellar surface. In addition, a higher degree of fragmentation was observed for Pt/NaNbO_3_, which may promote stronger metal–support interactions and consequently enhance the catalytic performance due to the beneficial role of the niobic acid support in improving the catalytic activity.^64^

**Figure 4:**
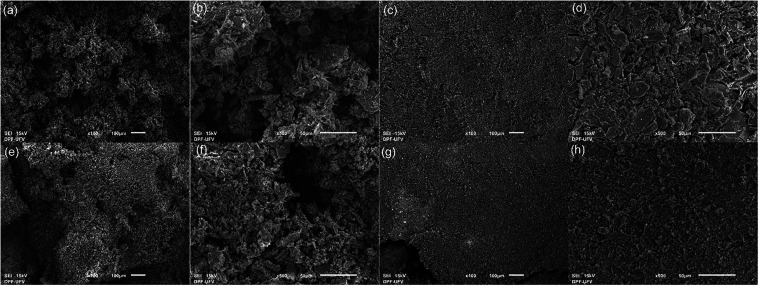
Scanning electron microscopy (SEM) images: (a, b) NaNbO_3_; (c, d) LiNbO_3_; (e, f) Pt NPs-NaNbO_3_; and (g, h) Pt NPs-LiNbO_3_.

Energy-dispersive X-ray spectroscopy (EDS) analysis (Figure S2), including elemental mapping (Figures S3–S6), was performed to identify the elements present in the materials, noting that lithium cannot be detected by EDS X-ray microanalysis. Accordingly, the expected elements (Nb, Na, and O) were observed for the support materials (Figure S2­(a,b)), as well as the presence of platinum in the prepared catalysts (Figure S2­(c,d)).

The materials were characterized by N_2_ adsorption/desorption, and the corresponding isotherms are shown in Figure S7. All samples (NaNbO_3_, LiNbO_3_, Pt NPs/NaNbO_3_, and Pt NPs/LiNbO_3_) exhibit Type IV­(a) isotherms, characteristic of mesoporous materials, where monolayer–multilayer adsorption is followed by capillary condensation at higher relative pressures.^65^ The textural parameters, including the surface area, pore volume, and average pore diameter, are summarized in Table 1. Comparable surface area values have been reported by Zhai et al.^66^ for niobium-based materials, with 3.91 m^2^ g^–1^ for LiNbO_3_ and 0.96 m^2^ g^–1^ for LiNb_3_O_8_. Interestingly, both NaNbO_3_ and LiNbO_3_ show an increase in surface area after Pt nanoparticle deposition. This behavior can be associated with the fact that the average particle size of Pt (6.51 and 6.57 nm) is larger than the mean pore diameters of the supports (3.84 and 3.08 nm), limiting pore insertion and favoring deposition on the external surface. As a result, surface roughness and external accessibility increase, contributing to the observed increase in surface area, in agreement with previous reports by de Souza et al.^67^ for Pt-decorated LiNbWO_6_ systems.

**Table 1:** Textural Properties of the Materials, Including the Specific Surface Area, Pore Volume, and Average Pore Diameter

materials	surface area (m^2^ g^–1^)	pore volume (cm^3^ g^–1^)	average pore diameter (nm)
NaNbO_3_	3.29	0.012	3.84
LiNbO_3_	8.77	0.017	3.08
Pt NPs/NaNbO_3_	6.77	0.024	3.85
Pt NPs/LiNbO_3_	10.07	0.019	3.84

Electrochemical impedance spectroscopy (EIS) was used to characterize NaNbO_3_ and LiNbO_3_ using the Fe­(CN)_6_^3–/4–^ redox probe. The Nyquist plots are shown in Figure S8. The charge transfer resistance (*R*_ct_) was estimated from the diameter of the semicircle in the high-frequency region, which reflects the ease of interfacial electron transfer; smaller semicircles indicate lower resistance and improved charge transfer kinetics.^68,69^ The *R*_ct_ values obtained were 462.5 Ω for NaNbO_3_ and 253.4 Ω for LiNbO_3_. The lower resistance observed for LiNbO_3_ indicates more favorable electron transfer at the electrode–electrolyte interface, suggesting higher electrochemical activity compared to NaNbO_3_.

### 2.2 Application of Pt NPs/MNbO_3_ in NaBH_4_ Hydrolysis

Different compositions of monometallic nanoparticles decorated on alkaline niobates were evaluated in the hydrolysis of NaBH_4_, and the results are shown in Figure 5. It can be observed that the support, without the nanoparticles, exhibited low kinetics and yield. However, Pt NPs/NaNbO_3_ (Figure 5(a)) exhibited higher kinetics compared to those of Pd NPs/NaNbO_3_, Co NPs/NaNbO_3_, and Ni NPs/NaNbO_3_ catalysts. Similar results were observed in Figure 5(b) with the use of Pt NPs/LiNbO_3_. This behavior may be associated with differences in metal–support interactions, nanoparticle dispersion, and surface properties of the synthesized catalysts under the evaluated reaction conditions.

**Figure 5:**
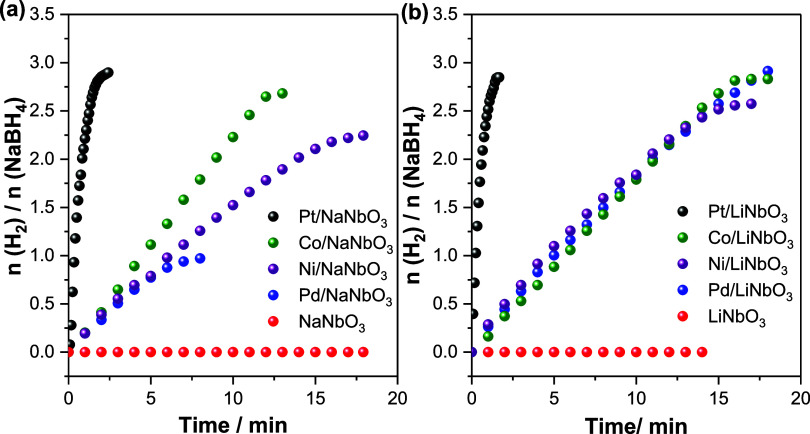
Hydrogen evolution from NaBH_4_ by metallic nanoparticles decorated on (a) NaNbO_3_ and (b) LiNbO_3_. Experimental conditions: 5 mg of support, 0.1067 mmol of catalyst, 1.0 mL of NaBH_4_ (0.500 mol L^–1^), constant agitation, and temperature of 298.15 K.

Under the same reaction conditions, Pt NPs/LiNbO_3_ demonstrated higher catalytic activity compared to Pt NPs/NaNbO_3_, as shown in Figure S9. This behavior can be attributed to the influence of dielectric properties on the catalytic activity between NaNbO_3_ and LiNbO_3_, resulting from differences in their cationic radii. Such variations in local coordination spheres align with efforts using nanoscale structural engineering to systematically lower activation barriers.^70^ Wang et al. compared LiNbO_3_, KNbO_3_, and NaNbO_3_, highlighting the differences in electronic and ionic transport mechanisms. In LiNbO_3_, mixed conduction (electronic and ionic) occurs, while in NaNbO_3_, Na^+^ ions are the predominant charge carriers. In LiNbO_3_, grain boundaries significantly impact the electronic transport process, whereas in NaNbO_3_, these boundaries do not contribute significantly to ionic conduction but instead generate charge polarization at the interface.^71^ This interpretation is further supported by electrochemical impedance spectroscopy (EIS) results, where LiNbO_3_ exhibited a lower charge transfer resistance (*R*_ct_ = 253.4 Ω) compared to NaNbO_3_ (*R*_ct_ = 462.5 Ω), indicating more favorable interfacial electron transfer. Such accelerated interfacial pathways are consistent with recent advancements in engineering ultrafast electron-transfer kinetics to overcome reaction mismatches.^72^ Therefore, the electronic–ionic conduction of LiNbO_3_ would account for the enhanced catalytic performance of the Pt nanoparticles supported on this material.

It is worth noting that the hydrolysis mechanism of NaBH_4_ involves the transfer of hydrogen to the nanoparticle. This phenomenon can occur via oxidative addition of a BH bond or through hydride transfer, with the negative charge dispersed across the surface.^73,74^ Furthermore, Figure S9 presents the catalytic performance of control experiments using unsupported Pt and a blank assay without a catalyst. No hydrogen evolution was observed in the absence of a catalyst, confirming the stability of NaBH_4_ under the experimental conditions. Furthermore, the unsupported Pt exhibited an approximately 20% decrease in the H_2_ production yield compared with the supported catalysts, highlighting the beneficial effect of the NaNbO_3_ and LiNbO_3_ supports on the hydrogen release efficiency.

The influence of sodium borohydride concentration on the rate of hydrogen generation was evaluated, and the results are shown in Figure S10­(a,b). The kinetic constants for each process were determined, and the plot of ln (k) versus ln (NaBH_4_ molar concentration) in Figure S11­(a,b) yields a slope of −0.33 ± 0.02 for Pt NPs/NaNbO_3_ and 1.17 ± 0.05 for Pt NPs/LiNbO_3_. These findings are indicative of zero-order and first-order kinetics for NaBH_4_ concentration when employing both catalysts, respectively.

The effect of catalyst dosage was evaluated in hydrogen evolution from NaBH_4_ for both NaNbO_3_ and LiNbO_3_ supports, as shown in Figure S12­(a,b). It was observed that as the catalyst quantity increases, the reaction rate also increases, suggesting that the hydrogen generation rate is proportional to the catalyst’s dosage. The graphic of ln (k) versus ln (catalyst dosage) in Figure S13­(a,b) results in a straight line with an approximate slope of 1.05 ± 0.11 for Pt NPs/NaNbO_3_ and 0.76 ± 0.02 for Pt NPs/LiNbO_3_, values close to unity. These results are consistent with first-order kinetics regarding the quantity of both catalysts. It is important to mention that the kinetic constant was determined for each catalyst considering different platinum (Pt) loadings deposited on the same mass of niobate (NaNbO_3_ or LiNbO_3_).

NaBH_4_ in an alkaline medium demonstrates high stability, justifying the investigation of the effect of NaOH concentration on hydrolysis. Additionally, it creates an electron-rich environment that can favor the formation of H_2_.^75^ The effect of NaOH concentration was evaluated, and the results are shown in Figure S14­(a,b). For Pt NPs/NaNbO_3_, low NaOH concentrations improved efficiency, with higher hydrogen production rates observed as the NaOH concentration decreased. In contrast, a concentration of 0.20 mol L^–1^ led to reduced efficiency. This effect was not observed for Pt NPs/LiNbO_3_, as NaOH did not improve the process efficiency. The graph of ln (*k*) versus ln (NaOH concentration) in Figure S15­(a,b) shows a straight line with an approximate slope of −0.33 ± 0.05 and −0.03 ± 0.003 for the use of Pt NPs/NaNbO_3_ and Pt NPs/LiNbO_3_ catalysts, respectively. This study observed a negative dependence at low concentrations, confirming the rate inhibition by NaOH. NaOH inhibits the reaction rate of the catalysts, resulting in a negative reaction order.

The slightly superior performance of Pt NPs/NaNbO_3_ in NaOH (0.01 mol L^–1^) can be attributed to enhanced ionic conductivity and the mitigation of undesirable effects, such as interfacial charge polarization.^71^ In contrast, the Pt NPs/LiNbO_3_ system showed a decline in performance under the same conditions. According to Didelhban et al.,^76^ hydroxyl ions compete with reactants for the catalyst’s active sites, leading to reduced catalytic activity. Additionally, the presence of NaOH can increase the viscosity of the reaction medium, hindering species diffusion and consequently lowering the hydrogen generation rate.

The effect of temperature on hydrogen evolution from NaBH_4_ using Pt NPs/NaNbO_3_ and Pt NPs/LiNbO_3_ was evaluated, and the results are shown in Figure S16. An increase in reaction kinetics was observed as the temperature rose. Arrhenius plots are shown in Figure 6(a,b), where the linear model allowed for the determination of the activation energies of both materials, according to eq 7. Activation energies of 35.54 ± 1.315 and 35.04 ± 1.319 kJ mol^–1^ were obtained for Pt NPs/NaNbO_3_ and Pt NPs/LiNbO_3_, respectively.

**Figure 6:**
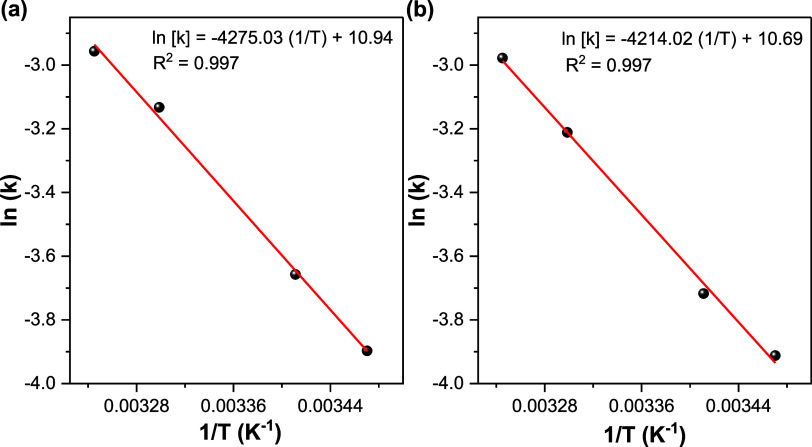
Arrhenius plots of hydrogen generation rates from NaBH_4_ by metallic nanoparticles decorated on (a) NaNbO_3_ and (b) LiNbO_3_. Experimental conditions: 5 mg of support, 0.1067 mmol of catalyst, 1.0 mL of NaBH_4_ (0.500 mol L^–1^), constant agitation.

The results obtained are comparable with the literature. For instance, multiwalled carbon nanotube-supported platinum (27 kJ mol^–1^) and carbon-supported platinum (36 kJ mol^–1^),^39^ Pt supported on Co_3_O_4_ (43.5 kJ mol^–1^),^77^ platinum-modified titanium dioxide support (53.2 kJ mol^–1^),^36^ highly dispersive platinum nanoparticles (39.2 kJ mol^–1^),^37^ poly­(ethylene glycol)-modified imidazolium monomer (23.9 kJ mol^–1^) and more active than its N-alkylated counterpart (35.6 kJ mol^–1^),^73^ and platinum nanoparticles supported on fused nanosized carbon spheres derived from sustainable sources (53 kJ mol^–1^).^40^ These comparisons underscore how advanced architectural designs in hybrid catalytic frameworks can effectively modulate energetic barriers and transport dynamics during hydrogen evolution pathways.^78^

Based on the results obtained at 308.15 K, models for determining the hydrogen generation rate (r_H_2__) for Pt/NaNbO_3_ NPs and Pt/LiNbO_3_ NPs were proposed, as shown in eqs 2 and 3, respectively

2 $$r_{H_{2}}=0.053{\left[\mathrm{NaB}H_{4}\right]}^{0.33}{\left[\mathrm{Pt NPs}/{\mathrm{NaNbO}}_{3}\right]}^{1.05}$$

3 $$r_{H_{2}}=0.050{\left[\mathrm{NaB}H_{4}\right]}^{1.17}{\left[\mathrm{Pt NPs}/{\mathrm{LiNbO}}_{3}\right]}^{0.76}$$

The catalysts Pt NPs/NaNbO_3_ and Pt NPs/LiNbO_3_ achieved hydrogen generation rates of 2044.9 and 2303.7 mL·min^–1^ g^–1^, respectively, at 293.15 K.

The kinetic isotope effect (KIE) for Pt NPs/NaNbO_3_ is presented in Figure 7a,b. A detailed understanding of the dehydrogenation mechanism is essential for the rational design of efficient catalysts, particularly regarding identifying the rate-determining step (RDS) in the reaction pathway. In this work, the KIE value (1.46) was used as a qualitative mechanistic probe, in line with its common application in heterogeneous catalysis and NaBH_4_ hydrolysis studies to indicate the involvement of hydrogen-related steps in the RDS, without requiring detailed mechanistic modeling.^79^ KIE is calculated as the ratio of rate constants for reactions involving light (k_H_) and heavy (k_D_) isotopically substituted reactants. As shown in Figure 7(b), the KIE H/D was determined to be 3.07/2.10 = 1.46. The reactions are classified into primary (KIE of 2–7) and secondary (KIE of 0.7–1.5) categories.^79^ A primary KIE indicates the breaking or formation of a bond containing this isotope in the slowest step of the reaction, whereas a secondary KIE suggests that the related bond is not affected in the rate-limiting stage. Such catalytic pathways necessitate a validation of functional mechanisms, as the synergistic interaction between active sites and support architectures directly governs the energetic barriers and molecular activation steps.^80^ In this sense, this analysis enables inference about which bonds undergo modification during the rate-limiting step of the reaction.^81^ Therefore, it can be concluded that the activation of water molecules on the surface of Pt/NaNbO_3_ NPs is not the determining step for the reaction.

**Figure 7:**
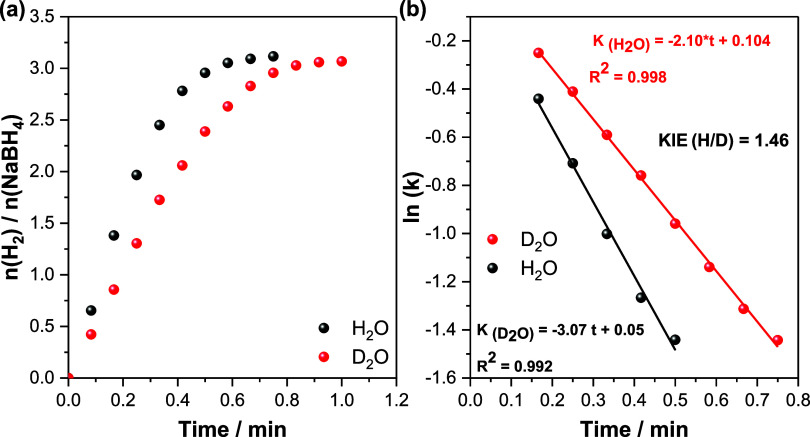
(a) Hydrogen evolution from the hydrolysis of NaBH_4_ in H_2_O and D_2_O, (b) kinetic isotope effect (KIE). Experimental conditions: 5 mg of support, 0.1067 mmol of catalyst, 1.0 mL of NaBH_4_ (0.500 mol L^–1^), volume: 2 mL, constant agitation, and temperature of 298.15 K.

The hydrolysis mechanism likely begins with Step I: the adsorption of BH_4_^–^ and H_2_O onto the catalyst surface.^81^ In Step II, a protic hydrogen from the adsorbed water interacts with a hydride hydrogen from the surface-bound borohydride species, forming a hydrogen bond that facilitates the oxidative addition of water by decreasing the electron density of the O–H bond. Subsequently, hydrogen is released via reductive elimination between metal-bound water and borohydride-derived hydride species. Notably, there is no evidence supporting direct cleavage of the O–H bonds of water; otherwise, the reaction would proceed significantly faster in H_2_O than in D_2_O.^73^

The reusability of the catalyst is a key parameter for potential industrial applications, and its stability and cyclic performance are essential for evaluating the practical applicability of functional materials.^82^ Therefore, reusability assays were conducted, and the results are shown in Figure 8(a,b). Notably, the material demonstrates a slight efficiency decrease of ∼20% only after the 10th cycle. This reduction in efficiency may be attributed to the precipitation of sodium metaborate (NaBO_2_) on the material’s surface, resulting in decreased accessibility to catalytic active sites.^83^ The loss of catalytic activity compared to the initial cycle indicates that the catalysts can be well reused up to 10–11 times. These results are comparable to the literature: For instance, platinum-modified titanium dioxide support (6 cycles),^36^ highly dispersive platinum nanoparticles (5 cycles),^37^ poly­(ethylene glycol)-modified imidazolium monomer (5 cycles),^73^ and platinum nanoparticles supported on fused nanosized carbon spheres (5 cycles).^40^ Furthermore, the incomplete attainment of the theoretical hydrogen yield may also be related to sodium metaborate accumulation and mass transfer limitations during the reaction, which can hinder the full hydrolysis of NaBH_4_ under practical operating conditions.

**Figure 8:**
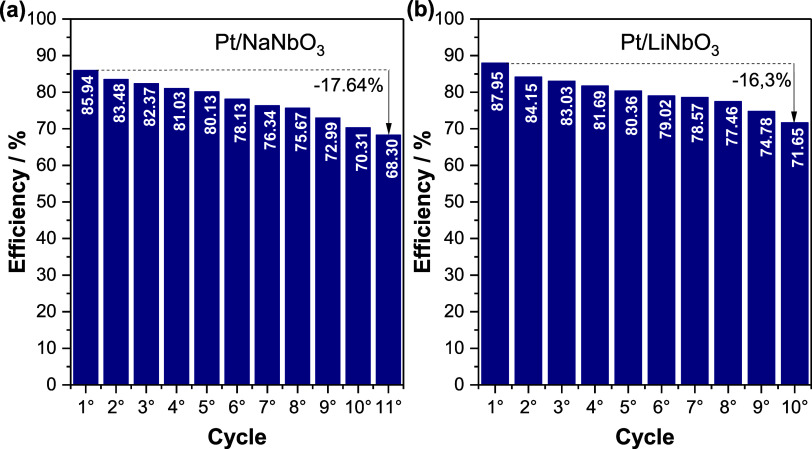
Reusability of the catalysts (a) Pt NPs/NaNbO_3_ and (b) Pt NPs/LiNbO_3_ in the hydrogen evolution from the hydrolysis of NaBH_4_. Experimental conditions: 5 mg of support, 0.1067 mmol of catalyst, 1.0 mL of NaBH_4_ (0.500 mol L^–1^), constant agitation, and temperature of 298.15 K.

The material after the catalytic use was characterized by different techniques. The presence of boron was detected in the reused material (Figures S2e,f and S17–S18), which may be attributed to the deposition of borate species on the catalyst surface. This surface coverage can directly hinder the interaction between the metallic nanoparticles and sodium borohydride during the subsequent hydrogen evolution cycles. Following catalyst reuse, the structure shows no visible changes, maintaining its initial appearance, as observed by SEM analysis (Figure S19). This suggests that the catalyst preserves its textural properties under reaction conditions.

ICP-OES analyses were performed on the supernatants from the first, second, and third reuse cycles to investigate possible Pt leaching and boron presence. Regarding Pt stability, no metal was detected in the supernatants of the Pt NPs/NaNbO_3_ material, while no detection was observed in the subsequent washing cycles for the Pt NPs/LiNbO_3_ material after the small initial fraction (0.05% of the total platinum loading) detected in the first cycle. Regarding boron, average amounts of 0.371 ± 0.023 mmol and 0.381 ± 0.002 mmol were identified in each washing for Pt NPs/LiNbO_3_ and Pt NPs/NaNbO_3_, respectively. Considering that 0.500 mmol of NaBH_4_ is added in each cycle, it is estimated that approximately 30% of the boron remains associated with the materials. This behavior is related to the formation and deposition of sodium metaborate on the catalyst surface, as evidenced in Figures S17 and S18, which may contribute to the decrease in catalytic efficiency during reuse cycles.

Table 2 compares the catalytic performances of different materials reported in the literature for hydrogen generation via NaBH_4_ hydrolysis. The Pt NPs/NaNbO_3_ and Pt NPs/LiNbO_3_ catalysts exhibited competitive hydrogen generation rate (HGR) values at relatively low temperatures, demonstrating the high catalytic efficiency of the proposed systems. In addition, the obtained activation energies were comparable to or lower than those reported for several catalysts in the literature. These comparisons highlight that integrating tailored compositional designs into alternative hydrogen evolution frameworks can effectively modulate energetic barriers and boost the overall H_2_ production efficiency.^84^

**Table 2:** Comparison of Catalytic Performance of Various Catalysts for Hydrogen Generation via NaBH_4_ Hydrolysis

catalyst	activation energy (kJ mol^–1^)	HGR (mL_H2_ min^–1^ g^–1^)	refs
MnCo-BDC^a^	12.40	1245.2 (27 °C)	^85^
Co–Cu–B^b^	49.60	734.4 (30 °C)	^86^
Pt/MWCNT^c^	27.00	1008.0 (27 °C)	^87^
Pt/Co_3_O_4_	43.50	4713.0 (25 °C)	^88^
Pd/Co_3_O_4_	65.80	2109.0 (25 °C)	^88^
Pt/TiO_2_	53.20	140.0 (30 °C)	^89^
Ru/TiO_2_	62.0	150.0 (30 °C)	^89^
Pt NPs/NaNbO_3_	35.54	2044.9 (20 °C)^d^	this work
Pt NPs/LiNbO_3_	35.04	2303.7 (20 °C)^d^	this Work

a Mn-BDC and MnCo-BDC (BDC—1,4-benzene-dicarboxylate) coordination polymers.

b Co–Cu–B catalysts prepared in methanol.

c Multiwalled carbon nanotube-supported platinum catalyst.

d The catalyst loading (mmol or wt %) was calculated based on the theoretical metal content, assuming complete incorporation of the precursor. ICP-OES quantification was not possible because of incomplete digestion of the samples under the available conditions.

## 3 Conclusion

The application of Pt NP/NaNbO_3_ and Pt NP/LiNbO_3_ nanocomposites for hydrogen generation through the hydrolysis of sodium borohydride solution is reported. A comparison between NaNbO_3_ and LiNbO_3_ in hydrogen evolution from NaBH_4_ was conducted for the first time. The synthesis of the materials, performed by using the Pechini method, was confirmed by various analytical techniques. LiNbO_3_ exhibited superior performance compared with NaNbO_3_, attributed to its enhanced electron–ionic conduction, which boosts the catalytic activity of platinum nanoparticles deposited on its surface. The catalyzed hydrolysis reaction exhibited first-order kinetics with respect to catalyst concentration for both systems while showing different dependencies on NaBH_4_ concentration, namely, near-zero-order for Pt NPs/NaNbO_3_ and first-order behavior for Pt NPs/LiNbO_3_, along with a near-zero-order dependence on NaOH concentration. Compared with several catalysts reported in the literature (Table 2), the Pt NPs/NaNbO_3_ and Pt NPs/LiNbO_3_ systems exhibited competitive hydrogen generation rates and relatively low activation energies, highlighting their potential as efficient catalysts for hydrogen production via NaBH_4_ hydrolysis. Moreover, both catalysts, in addition to being cost-effective, demonstrated high recyclability. Their catalytic activities showed a moderate decrease of less than 20% after multiple cycles while still maintaining satisfactory performance, reinforcing the environmentally sustainable nature of the process. Thus, this robust catalytic system presents itself as an economically viable and highly efficient solution for the alkaline hydrolysis of sodium borohydrides.

## 4 Experimental Section

### 4.1 Standards and Reagents

All reagents used were analytical grade. Sodium borohydride 98% (CAS 16940-66-2), cobalt nitrate hexahydrate 98% (CAS 10026-22-9), and sodium hydroxide 95% (CAS 1310-73-2) were purchased from Vetec. Ethylene glycol 99.5% (CAS 107-21-1), citric acid 99% (CAS 77-92-9), hexachloroplatinic acid hexahydrate 99.9% (CAS 18497-13-7), lithium nitrate 99.9% (CAS 7790-69-4), sodium nitrate 99% (CAS 7631-99-4), niobium ammonium oxalate 99.99% (CAS 168547-43-1), nickel sulfate heptahydrate 98% (CAS 10101-97-0), potassium tetrachloropalladate (II) 98% (CAS 10025-98-6), and hexachloroplatinic (IV) acid hexahydrate 99.9% (CAS 18497-13-7) were purchased from Sigma-Aldrich. All solutions were prepared using type 1 water, obtained in a Milli-Q system from Merck Millipore.

### 4.2 Synthesis of MNbO_3_ (M = Na, Li)

NaNbO_3_ and LiNbO_3_ were synthesized using the modified Pechini method.^90,91^ In this procedure, two beakers were utilized simultaneously. Beaker 1 contained a solution of NaNO_3_ or LiNO_3_ at a concentration of 0.100 mol L^–1^, while beaker 2 contained a solution of niobium ammoniacal oxalate at the same concentration, both maintained at a temperature of 50 °C. Subsequently, 30 mmol of citric acid was added to each beaker, and the temperature was maintained at 50 °C for 30 min. Following this, the temperature was raised to 90 °C, and 2.1 mL of ethylene glycol was added, with the temperature being maintained at 90 °C for an additional 30 min. Upon completion of this period, the contents of beaker 1 were slowly poured into beaker 2 and held at 90 °C for another 30 min. Subsequently, the polymeric resin containing the metallic citrate was heated in an oven for 12 h at 115 °C, pulverized in a mortar, heated in a muffle furnace for 2 h at 600 °C (with a heating rate of 2 °C min^–1^), cooled to room temperature (∼25 °C), washed with 50 mL of Milli-Q water, and centrifuged at 4000 rpm for 10 min, agitated on a vortex mixer for 1 min, repeating the washing process twice.

### 4.3 Preparation of MNbO_3_ (M = Na, Li) Decorated with Metallic Nanoparticles

Monometallic nanoparticles (Ni, Co, Pt and Pd NPs) decorated on NaNbO_3_ or LiNbO_3_ were prepared according to Sperandio et al.^10^ For this purpose, 5 mg of NaNbO_3_ or LiNbO_3_ was dispersed in 5 mL of type 1 water in a beaker under stirring (180 rpm). Subsequently, 0.1067 mmol of each metal precursor salt, NiSO_4_·7H_2_O, Co­(NO_3_)_2_·6H_2_O, H_2_PtCl_6_·6H_2_O, or K_2_PdCl_4_, was added to the system, which was stirred for 15 min at room temperature (25 °C). Then, 1.00 mL of an excess NaBH_4_ solution (1.0 mol L^–1^) was added to the system for the ex situ reduction process, which was stirred for an additional 15 min to ensure complete reduction of the catalyst metals, thereby guaranteeing the desired concentration of nanoparticles. The suspension was centrifuged at 4000 rpm for 15 min and washed three times with type 1 water. Freshly prepared material was used in the hydrogen evolution. The deposition of Pt nanoparticles was carried out immediately prior to catalytic testing in order to minimize potential oxidation effects. The pH of the reaction system corresponded to the natural pH of the prepared solution, without further adjustment.

### 4.4 Catalyst Characterization

Advanced analytical approaches have been increasingly employed for detailed compositional characterization of complex systems.^92^ In this context, the physicochemical properties of the prepared materials were investigated using a combination of complementary characterization techniques. The phases of the materials were determined by powder X-ray diffraction (XRD) patterns, collected in a Shimadzu Diffractometer model XRD-7000, with a current of 30 mA and a voltage of 30 kV, using Cu Kα radiation (λ = 1.541838 Å). The analysis was conducted in the 2θ range from 10° to 80° with a scanning rate of 1°/min. The patterns obtained were compared to those data deposited at JCPDS (International Centre for Diffraction Data). The XRD results obtained were used to calculate the crystallite size of the NaNbO_3_ and LiNbO_3_ materials through Scherrer analysis.

Scanning electron microscopy (SEM) imaging was conducted with a JEOL JSM-6010LA scanning electron microscope, providing a resolution of 4 nm and operating at a voltage of 20 kV. Magnifications ranged from 5× to 300,000×, with the acceleration voltage varying between 500 V and 20 kV, and a precentered tungsten filament electron gun. High-resolution transmission electron microscopy (HRTEM) analysis was carried out using a JEM-2100 Jeol Thermo Scientific microscope. Such advanced nanoscale characterization perspectives are critical for elucidating dynamic structure–property relationships in tailored catalytic systems.^93^

The specific surface area (SSA) by nitrogen adsorption method was developed by Brunauer–Emmett–Teller (BET) using a Micrometric ASAP 2020. Before the measurements, the sample was vacuum-dried at 130 °C for 48 h to eliminate any remaining water and gases.

Electrochemical measurements were carried out using a μStat-I 400s BiPotentiostat/Galvanostat/Impedance Analyzer (Metrohm DropSens, USA). Electrochemical impedance spectroscopy (EIS) measurements were carried out using the ferricyanide/ferrocyanide redox couple in 0.1 mol L^–1^ KCl solution containing 5.0 mmol L^–1^ of K_3_Fe­(CN)_6_ and K_4_Fe­(CN)_6_.

The platinum and boron contents were determined by inductively coupled plasma optical emission spectrometry (ICP-OES) using Thermo Scientific i-CAP PRO equipment at wavelengths of 224 and 208 nm, respectively.

### 4.5 General Procedure for Hydrogen Evolution

The freshly prepared catalyst was dispersed in 10.0 mL of Milli-Q water within a Kitasato apparatus, which was then sealed with a rubber septum and attached to a buret containing water. Next, 1.00 mL of a NaBH_4_ solution at the desired concentration was injected by using a syringe through the rubber septum. The system was continuously stirred while maintaining the controlled temperature conditions. The volume of hydrogen produced was determined by the displacement of water in the buret.

The pressure (*P*) exerted by the generated hydrogen gas within a buret was determined according to eq 4, considering the following parameters: ρ (water density) = 1000 kg m^–3^, g (acceleration due to gravity) = 9.78 m s^2^, local atmospheric pressure of 942,589 Pa (707 mmHg), and the observed displacement (*h*), measured in meters. The absolute pressure was adjusted as per eq 4

4 $$P=P_{0}+\rho \mathrm{gh}$$

### 4.6 Hydrogen Generation Rate (HGR)

The hydrogen generation rate (HGR) was calculated according to eq 5

5 $$\mathrm{HGR}=\frac{{\Delta V}_{H_{2}}}{\Delta t\times m_{\mathrm{cat}}}$$

where Δ*V*_H_2__ is the hydrogen volume variation in mL, Δ*t* is the time variation in min, and *m*_cat_ is the catalyst mass in grams.

### 4.7 Kinetic Studies

The kinetics of sodium borohydride hydrolysis using Pt NPs decorated on MNbO_3_ (M = Na, Li) were examined by evaluating various parameters, including (i) catalyst dosage, (ii) NaOH concentration, (iii) NaBH_4_ concentration, and (iv) temperature.

#### 4.7.1. Catalyst Dose Effect

The hydrogen evolution was carried out using different doses of catalyst Pt NPs (0.0133, 0.0266, 0.0532, and 0.1064 mmol). The other parameters were kept constant, including 5 mg of MNbO_3_, 1.00 mL of NaBH_4_ (0.500 mol L^–1^), Pt/NaBH_4_ ratio (0.0266, 0.0532, 0.1064, 0.2128 mmol Pt/mmol NaBH_4_), constant agitation, and a temperature of 298.15 K.

#### 4.7.2. Effect of NaOH Concentration

Hydrogen evolution was conducted using NaBH_4_ (0.500 mol L^–1^) dissolved in 1.00 mL of NaOH solution under stirring. The NaOH solution was prepared at different concentrations (0.010, 0.050, 0.100, and 0.200 mol L^–1^). The other parameters were maintained constant, including 5 mg of MNbO_3_ (M = Na, Li) containing 0.1067 mmol (0.021 g) of Pt, constant agitation, and a temperature of 298.15 K.

#### 4.7.3. Effect of NaBH_4_ Concentration

The hydrogen evolution was performed using 1.00 mL of NaBH_4_ at various concentrations (0.350, 0.400, 0.450, and 0.500 mol L^–1^). The other parameters were maintained constant, including 5 mg of MNbO_3_ (M = Na, Li) containing 0.1067 mmol (0.021 g) of Pt, constant agitation, and a temperature of 298.15 K.

#### 4.7.4. Temperature Effect

The effect of temperature was also evaluated with temperatures tested at 288.15, 293.15, 303.15, and 308.15 K. The other parameters were kept constant, including 5 mg of MNbO_3_ (M = Na, Li) containing 0.1067 mmol (0.021 g) of Pt, 1.0 mL of NaBH_4_ (0.500 mol L^–1^), constant agitation, and a temperature of 293.15 K.

### 4.8 Kinetic Isotope Effect (KIE) Assay

To investigate the mechanism of the catalytic reaction, the Pt NP/NaNbO_3_ catalyst was first synthesized, washed with acetone, and then dried under vacuum. The catalyst (5 mg of NaNbO_3_ containing 0.021 g of Pt) was then placed in a 10 mL Schlenk flask. After sealing the flask, it was connected to a buret filled with water. Subsequently, 1.0 mL of a freshly prepared solution containing 0.500 mol L^–1^ of NaBH_4_ in deuterated water (D_2_O) or type 1 water was introduced using a syringe. All of the reactions were conducted at 298.15 K.

### 4.9 Hydrogen Evolution Rate and Activation Energy

The hydrogen evolution rate can be related to temperature, concentrations of NaBH_4_, Pt NPs/MNbO_3_ dosage, and NaOH concentration, according to eq 6

6 $$r_{H_{2}}=-4\frac{d\left[\mathrm{NaB}H_{4}\right]}{dt}=\frac{d\left[H_{2}\right]}{dt}=k_{\mathrm{overall}}{\left[\mathrm{NaB}H_{4}\right]}^{a}{\left[\mathrm{Pt NPs}/\mathrm{MNb}O_{3}\right]}^{b}{\left[\mathrm{NaOH}\right]}^{c}$$

wherein *a*, *b* and *c* are the orders of the reaction.

The activation energy was calculated using the Arrhenius equation, eq 7

7 $$\ln\left(k_{\mathrm{overall}}\right)=\ln\left(A\right)-\frac{E_{a}}{\mathrm{RT}}$$

wherein *k*_overall_ is the kinetic constant of the reaction, *A* is the pre-exponential factor, *E*_a_ is the apparent activation energy (in kJ mol^–1^), *R* is the universal gas constant, and *T* is the temperature in Kelvin.

### 4.10 Reuse of the Catalyst

After each cycle, the resulting suspension was centrifuged (4000 rpm, 15 min), the supernatant was removed, and the solid was washed with 30 mL of type 1 water. This washing procedure was repeated twice with Milli-Q water. The recovered solid was then dispersed in 10.0 mL of type 1 water and reintroduced into the Kitasato for another cycle. The excellent sedimentation and decantation behavior of the material minimized catalyst loss during the washing and recovery procedures. This process was repeated until a ∼20% reduction in hydrogen generation efficiency was observed. The utilized conditions comprised 5 mg of MNbO_3_ (M = Na, Li) containing 0.1067 mmol (0.021 g) of Pt, 1.0 mL of NaBH_4_ (0.500 mol L^–1^), constant agitation, and a temperature of 298.15 K.

Catalytic experiments were performed in sequential runs under identical conditions for the same support batch, following a standardized protocol to ensure data consistency and reproducibility. All experimental data were compiled and processed using Microsoft Excel (Microsoft Office 365), and graphical representations were generated using OriginPro 2021 software.

## Supplementary Material



## Data Availability

All data are available in the text.

## Notes

SEM images of synthesized LiNbO_3_ at magnifications of (a) 5k×, (b) 25k×, and (c) 50k× and images of synthesized NaNbO_3_ at magnifications of (d) 5k×, (e) 25k×, and (f) 50k× (Figure S1); energy-dispersive X-ray spectra (EDS) of the materials: (a) NaNbO_3_, (b) LiNbO_3_, (c) Pt NPs-NaNbO_3_, (d) Pt NPs-LiNbO_3_, and (e) Pt NPs-NaNbO_3_ after catalyst reusability, and (f) Pt NPs-LiNbO_3_ after catalyst reusability (Figure S2); elemental mapping of NaNbO_3_ obtained through energy-dispersive X-ray spectroscopy (EDS) analysis (Figure S3); elemental mapping of LiNbO_3_ obtained through energy-dispersive X-ray spectroscopy (EDS) analysis (Figure S4); elemental mapping of Pt NPs-NaNbO_3_ obtained through energy-dispersive X-ray spectroscopy (EDS) analysis (Figure S5); elemental mapping of Pt NPs-LiNbO_3_ obtained through energy-dispersive X-ray spectroscopy (EDS) analysis (Figure S6); nitrogen adsorption and desorption isotherms of the materials: (a) NaNbO_3_, (b) LiNbO_3_, (c) Pt NPs/NaNbO_3_, and (d) Pt NPs/LiNbO_3_ (Figure S7); Nyquist plots recorded for NaNbO_3_ and LiNbO_3_ electrodes in KCl solution (0.1 mol L^–1^) containing K_3_Fe­(CN)_6_/K_4_Fe­(CN)_6_ (5.0 mmol L^–1^) redox couple­(Figure S8); hydrogen evolution from NaBH_4_ using platinum nanoparticles supported on NaNbO_3_ and LiNbO_3_, unsupported platinum, and a blank hydrolysis control without catalyst; experimental conditions: 5 mg of support, 0.1067 mmol of catalyst, 1.0 mL of NaBH_4_ (0.500 mol L^–1^), constant agitation, and temperature of 298.15 K (Figure S9); hydrogen evolution from different NaBH_4_ concentrations by metallic nanoparticles decorated on (a) NaNbO_3_ and (b) LiNbO_3_; experimental conditions: 5 mg of support, 0.1067 mmol of catalyst, 1.0 mL of NaBH_4_ (0.500 mol L^–1^), constant agitation, and temperature of 298.15 K (Figure S10); ln (k) vs ln (NaBH_4_ molar concentration) of (a) Pt NPs/NaNbO_3_ and (b) Pt NPs/LiNbO_3_; experimental conditions: 5 mg of support, 0.1067 mmol of Pt, 1.0 mL of NaBH_4_ (0.500 mol L^–1^), constant agitation, and temperature of 298.15 K (Figure S11); hydrogen evolution from the hydrolysis of NaBH_4_ at different catalyst dosages (0.0133, 0.0267, 0.0533, and 0.1067 mmol of Pt): (a) Pt NPs/NaNbO_3_ and (b) Pt NPs/LiNbO_3_; experimental conditions: 5 mg of support, 1.0 mL of NaBH_4_ (0.500 mol L^–1^), constant agitation, and temperature of 298.15 K (Figure S12); ln (k) vs ln (catalyst dosage) of (a) Pt NPs/NaNbO_3_ and (b) Pt NPs/LiNbO_3_; experimental conditions: 5 mg of support, 0.1067 mmol of Pt, 1.0 mL of NaBH_4_ (0.500 mol L^–1^), constant agitation, and temperature of 298.15 K (Figure S13); hydrogen evolution from the hydrolysis of NaBH_4_ at different NaOH concentrations using (a) Pt NPs/NaNbO_3_ and (b) Pt NPs/LiNbO_3_; experimental conditions: 5 mg of support, 0.1067 mmol of catalyst, 1.0 mL of NaBH_4_ (0.500 mol L^–1^), constant agitation, and temperature of 298.15 K (Figure S14); ln (k) vs ln (NaOH molar concentration) of (a) Pt NPs/NaNbO_3_ and (b) Pt NPs/LiNbO_3_; experimental conditions: 5 mg of support, 0.1067 mmol of Pt, 1.0 mL of NaBH_4_ (0.500 mol L^–1^) in NaOH solutions, constant agitation, and temperature of 298.15 K (Figure S15); evaluation of temperature in hydrogen evolution from NaBH_4_ by (a) Pt NPs/NaNbO_3_ and (b) Pt NPs/LiNbO_3_; experimental conditions: 5 mg of support mass, 0.1067 mmol of Pt, 1.0 mL of NaBH_4_ (0.500 mol L^–1^), constant agitation (Figure S16); elemental mapping of Pt NPs-NaNbO_3_ after the catalyst reusability obtained through energy-dispersive X-ray spectroscopy (EDS) analysis (Figure S17); elemental mapping of Pt NPs-LiNbO_3_ after the catalyst reusability obtained through energy-dispersive X-ray spectroscopy (EDS) analysis (Figure S18); and scanning electron microscopy (SEM) images: (a,b) Pt NPs-NaNbO_3_ after catalyst reusability and (c,d) Pt NPs-LiNbO_3_ after catalyst reuse, with 100× and 500× magnifications, respectively (Figure S19) (PDF)
