# Supplementary material for: Platinum Nanoparticles Decorated on NaNbO3 and LiNbO3 as Catalysts for Enhanced Hydrogen Generation via NaBH4 Hydrolysis
Source: ACS Omega. 2026 Jun 13;11(25):37904–18. doi: 10.1021/acsomega.6c02694 (PMC13325092; doi:10.1021/acsomega.6c02694)
Supplement: Supplementary file 1 [file ao6c02694_si_001.pdf]

Platinum Nanoparticles Decorated on $NaNbO_3$ and $LiNbO_3$ as Catalysts for Enhanced Hydrogen Generation via $NaBH_4$ Hydrolysis

Iterlandes Machado Junior[a], Matheus Araújo Pereira[b], Gabriel Henrique Sperandio[a], Kleryton Luiz Alves de Oliveira[a], Antonio Machado Netto[a], Fabrício Vieira de Andrade[b] and Renata Pereira Lopes Moreira[a]*.

[a] *Department of Chemistry, Universidade Federal de Viçosa (UFV), Av. Peter Henry Rolfs, s/n, Campus Universitário, 36.570-000, Viçosa, Minas Gerais, Brazil*

[b] *Universidade Federal de Itajubá (UNIFEI), Campus Itabira, Rua Irmã Ivone Drumond, 200, Distrito Industrial II, 35903087, Itabira, Minas Gerais, Brazil*

*Corresponding author:

E-mail address: renata.plopes@ufv.br; Telephone: +55-31-3612-6676

E-mail address: fabricio.andrade@unifei.edu.br; Telephone: +55-31- 31 3839-0897

Sumário

Figure S1. SEM images of synthesized LiNbO3 at magnifications of (a) 5kX, (b) 25kX, and(c) 50kX; and images of synthesized NaNbO3 at magnifications of (d) 5kX, (e) 25kX, and(f) 50kX. 4

Figure S2. Energy-dispersive X-ray spectra (EDS) of the of the materials. (a) $NaNbO_3$; (b) $LiNbO_3$; (c) Pt NPs-$NaNbO_3$; (d) Pt NPs-$LiNbO_3$; (e) Pt NPs-$NaNbO_3$ after the catalyst reusability; (f) Pt NPs-$LiNbO_3$ after the catalyst reusability....4

Figure S3. Elemental mapping of $NaNbO_3$ obtained through energy-dispersive X-ray spectroscopy (EDS) analysis....5

Figure S4. Elemental mapping of $LiNbO_3$ obtained through energy-dispersive X-ray spectroscopy (EDS) analysis....5

Figure S5. Elemental mapping of Pt NPs-$NaNbO_3$ obtained through energy-dispersive X-ray spectroscopy (EDS) analysis....6

Figure S6. Elemental mapping of Pt NPs-$LiNbO_3$ obtained through energy-dispersive X-ray spectroscopy (EDS) analysis....6

Figure S7. Nitrogen adsorption and desorption isotherms of the materials. (a) $NaNbO_3$; (b) $LiNbO_3$; (c) Pt NPs/$NaNbO_3$; (d) Pt NPs/$LiNbO_3$ 7

Figure S9. Hydrogen evolution from $NaBH_4$ using platinum nanoparticles supported on $NaNbO_3$ and $LiNbO_3$, unsupported platinum, and a blank hydrolysis control without catalyst. Experimental conditions: 5 mg of support, 0.1067 mmol of catalyst, 1.0 mL $NaBH_4$ (0.500 mol L^{-1}), constant agitation, and temperature of 298.15 K. 8

Figure S10. Hydrogen evolution from different NaBH4 concentration by metallic nanoparticles decorated on (a) NaNbO3 and (b) LiNbO3. Experimental conditions: 5 mg of support, 0.1067 mmol of catalyst, 1.0 mL NaBH4 (0.500 mol L-1), constant agitation, and temperature of 298.15 K. 8

Figure S11. ln (k) *vs* ln ($NaBH_4$ molar concentration) of (a) Pt NPs/$NaNbO_3$ and (b) Pt NPs/$LiNbO_3$. Experimental conditions: 5 mg of support, 0.1067 mmol of Pt, 1.0 mL $NaBH_4$ (0.500 mol L^{-1}), constant agitation, and temperature of 298.15 K....9

Figure S12. Hydrogen evolution from the hydrolysis of $NaBH_4$ at different catalyst dosages (0.0133, 0.0267, 0.0533 and 0.1067 mmol Pt). (a) Pt NPs/$NaNbO_3$ and (b) Pt NPs/$LiNbO_3$. Experimental conditions: 5 mg of support, 1.0 mL $NaBH_4$ (0.500 mol L^{-1}), constant agitation, and temperature of 298.15 K....9

Figure S13. ln (k) *vs* ln (catalyst dosage) of (a) Pt NPs/$NaNbO_3$ and (b) Pt NPs/$LiNbO_3$. Experimental conditions: 5 mg of support, 0.1067 mmol of Pt, 1.0 mL $NaBH_4$ (0.500 mol L^{-1}), constant agitation, and temperature of 298.15 K....10

Figure S14. Hydrogen evolution from the hydrolysis of $NaBH_4$ at different NaOH concentration using (a) Pt NPs/$NaNbO_3$ and (b) Pt NPs/$LiNbO_3$. Experimental conditions: 5 mg of support, 0.1067 mmol of catalyst, 1.0 mL $NaBH_4$ (0.500 mol L^{-1}), constant agitation, and temperature of 298.15 K....10

Figure S15. ln (k) *vs* ln (NaOH molar concentration) of (a) Pt NPs/$NaNbO_3$ and (b) Pt NPs/$LiNbO_3$. Experimental conditions: 5 mg of support, 0.1067 mmol of Pt, 1.0 mL $NaBH_4$ (0.500 mol L^{-1}) in NaOH solutions, constant agitation, and temperature of 298.15 K.........11

Figure S16. Evaluation of temperature in hydrogen evolution from $NaBH_4$ by (a) Pt NPs/$NaNbO_3$ and (b) Pt NPs/$LiNbO_3$. Experimental conditions: 5 mg of support mass, 0.1067 mmol of Pt, 1.0 mL $NaBH_4$ (0.500 mol L^{-1}), constant agitation..............................11

Figure S17. Elemental mapping of Pt NPs-$NaNbO_3$ after the catalyst reusability obtained through energy-dispersive X-ray spectroscopy (EDS) analysis. 12

Figure S18. Elemental mapping of Pt NPs-$LiNbO_3$ after the catalyst reusability obtained through energy-dispersive X-ray spectroscopy (EDS) analysis. 12

Figure S19. Scanning Electron Microscopy (SEM) images: (a) and (b) Pt NPs-$NaNbO_3$ after the catalyst reusability; (c) and (d) Pt NPs-$LiNbO_3$ after the catalyst reuse, with 100× and 500× magnifications, respectively. 13

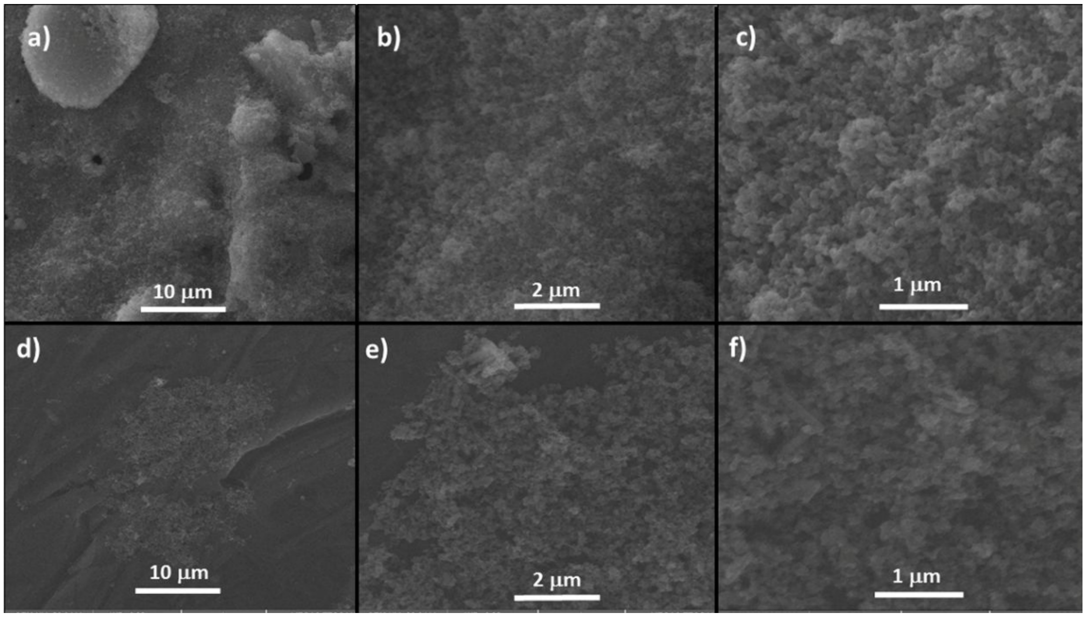

Figure S1. SEM images of synthesized LiNbO3 at magnifications of (a) 5kX, (b) 25kX, and(c) 50kX; and images of synthesized NaNbO3 at magnifications of (d) 5kX, (e) 25kX, and(f) 50kX.

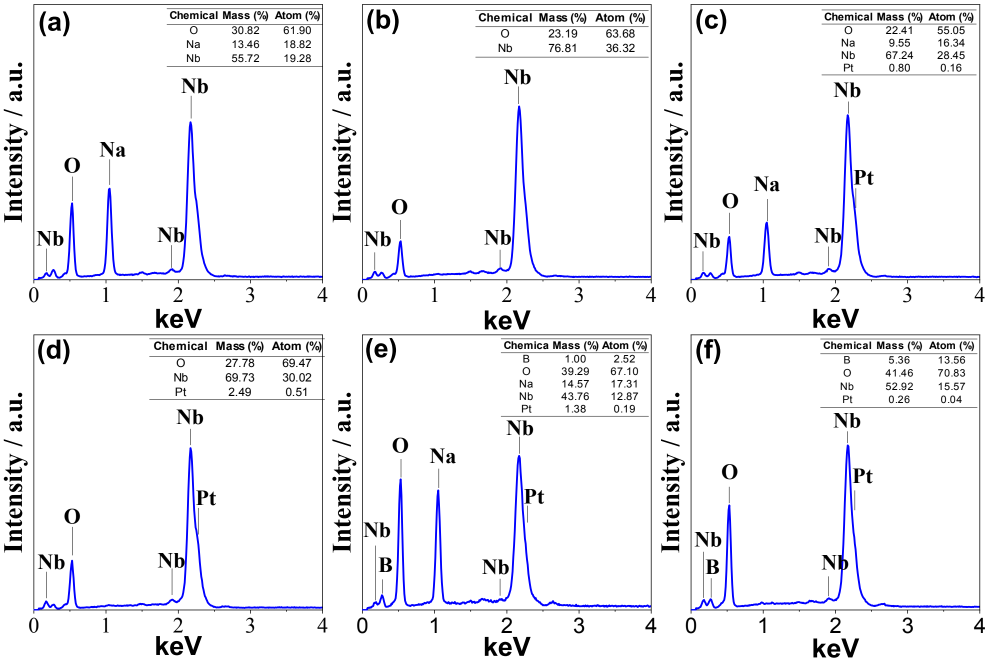

Figure S2. Energy-dispersive X-ray spectra (EDS) of the of the materials. (a) $NaNbO_3$; (b) $LiNbO_3$; (c) Pt NPs-$NaNbO_3$; (d) Pt NPs-$LiNbO_3$; (e) Pt NPs-$NaNbO_3$ after the catalyst reusability; (f) Pt NPs-$LiNbO_3$ after the catalyst reusability.

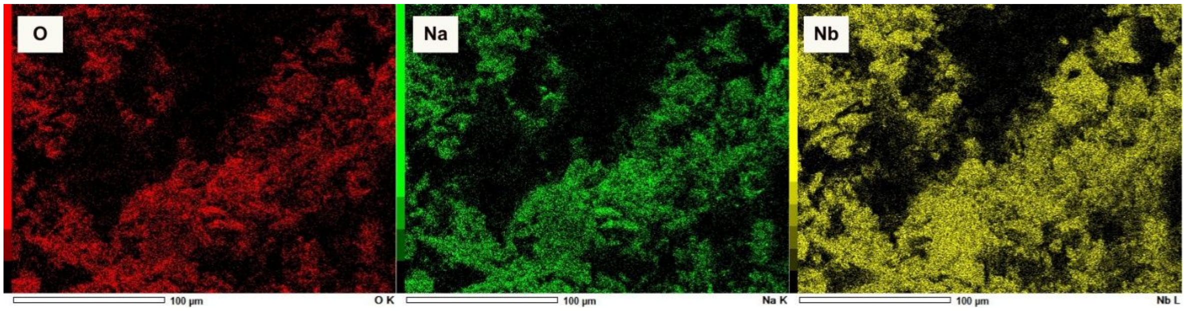

Figure S3. Elemental mapping of $NaNbO_3$ obtained through energy-dispersive X-ray spectroscopy (EDS) analysis.

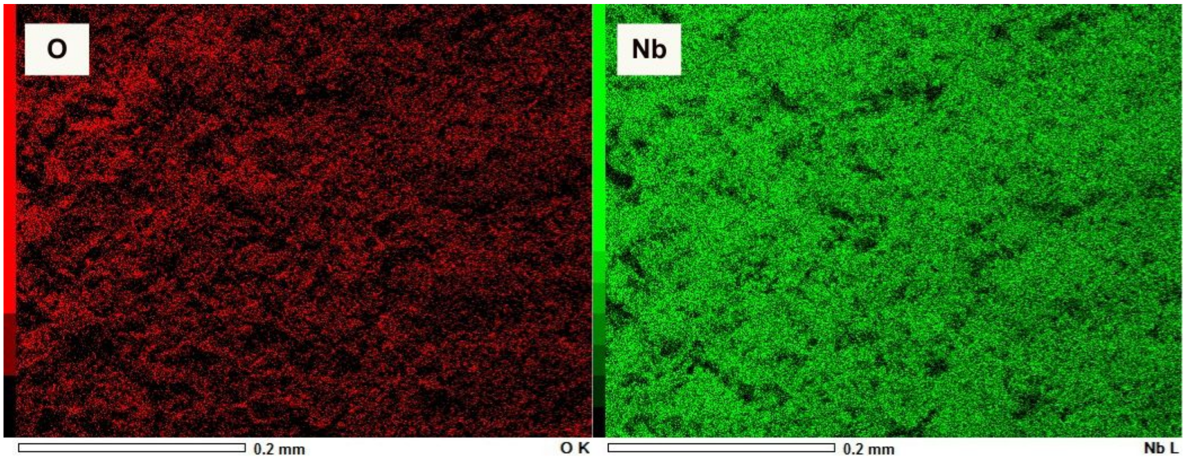

Figure S4. Elemental mapping of $LiNbO_3$ obtained through energy-dispersive X-ray spectroscopy (EDS) analysis.

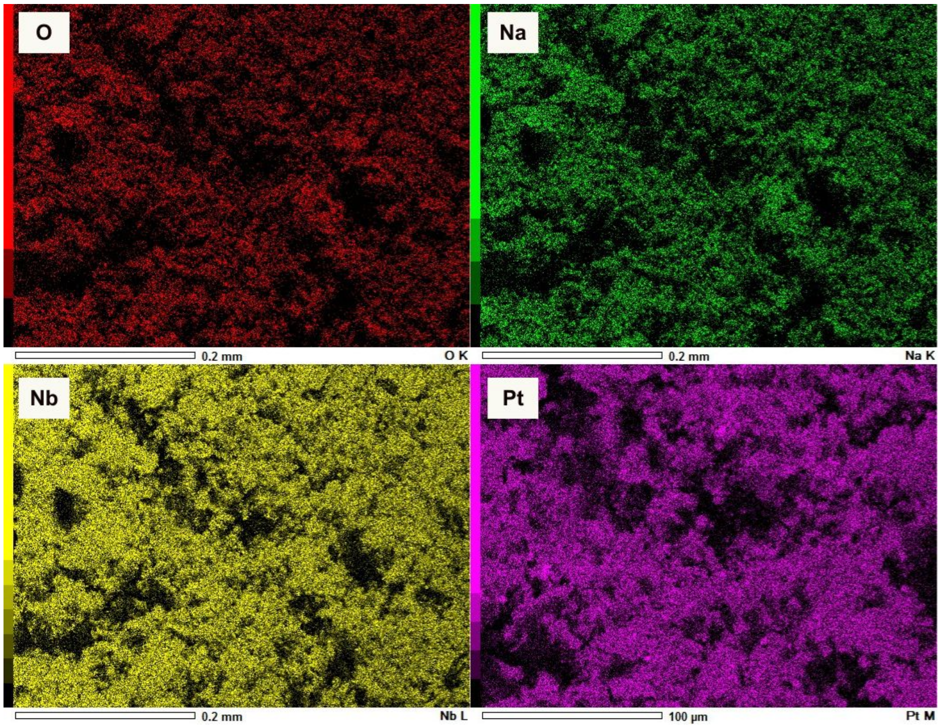

Figure S5. Elemental mapping of Pt NPs-$NaNbO_3$ obtained through energy-dispersive X-ray spectroscopy (EDS) analysis.

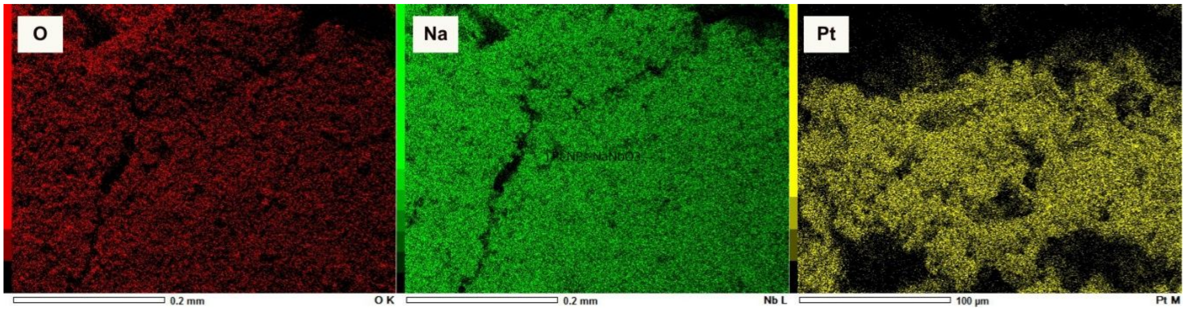

Figure S6. Elemental mapping of Pt NPs-$LiNbO_3$ obtained through energy-dispersive X-ray spectroscopy (EDS) analysis.

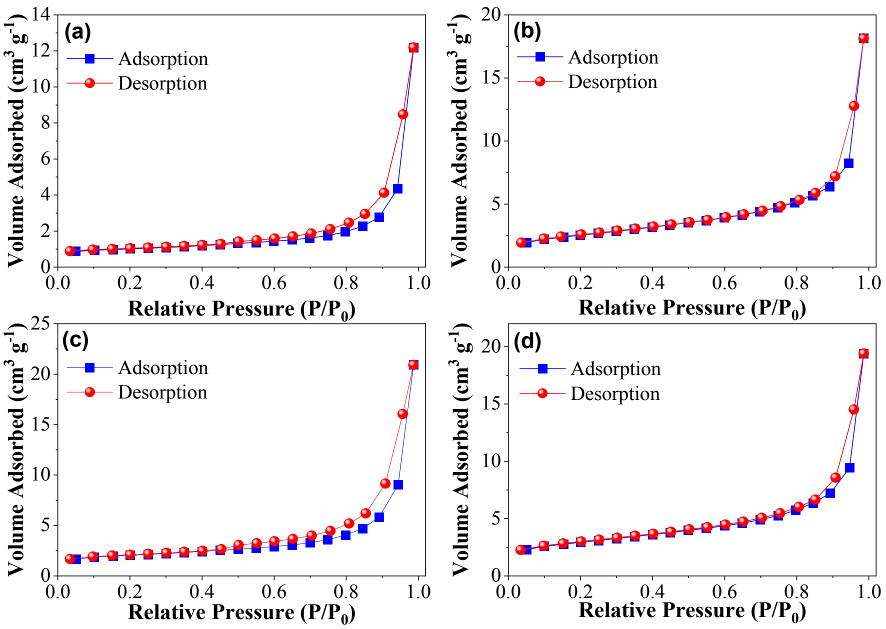

Figure S7. Nitrogen adsorption and desorption isotherms of the materials. (a) $NaNbO_3$; (b) $LiNbO_3$; (c) Pt NPs/$NaNbO_3$; (d) Pt NPs/$LiNbO_3$

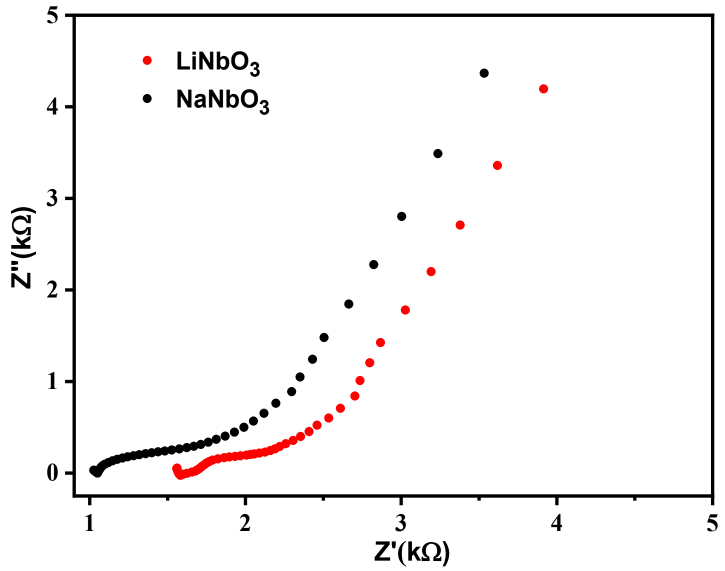

,

Figure S8. Nyquist plots recorded for $NaNbO_3$ and $LiNbO_3$ electrodes in KCl solution (0.1 mol L^{-1}) containing $K_3Fe(CN)_6/K_4Fe(CN)_6$ (5.0 mmol L^{-1}) redox couple.

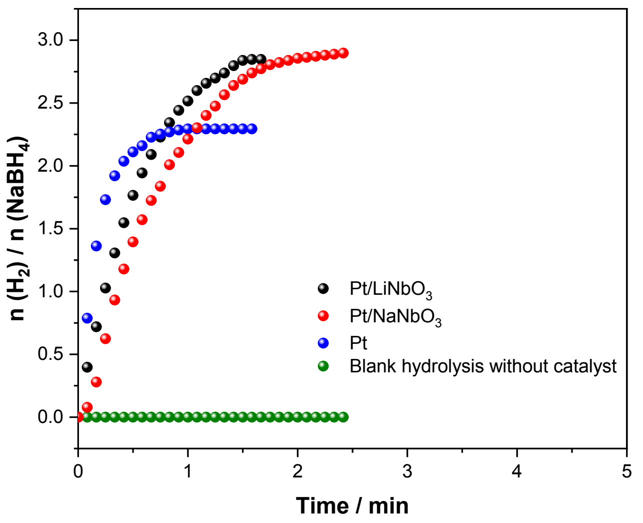

Figure S9. Hydrogen evolution from $NaBH_4$ using platinum nanoparticles supported on $NaNbO_3$ and $LiNbO_3$, unsupported platinum, and a blank hydrolysis control without catalyst. Experimental conditions: 5 mg of support, 0.1067 mmol of catalyst, 1.0 mL $NaBH_4$ (0.500 mol L^{-1}), constant agitation, and temperature of 298.15 K.

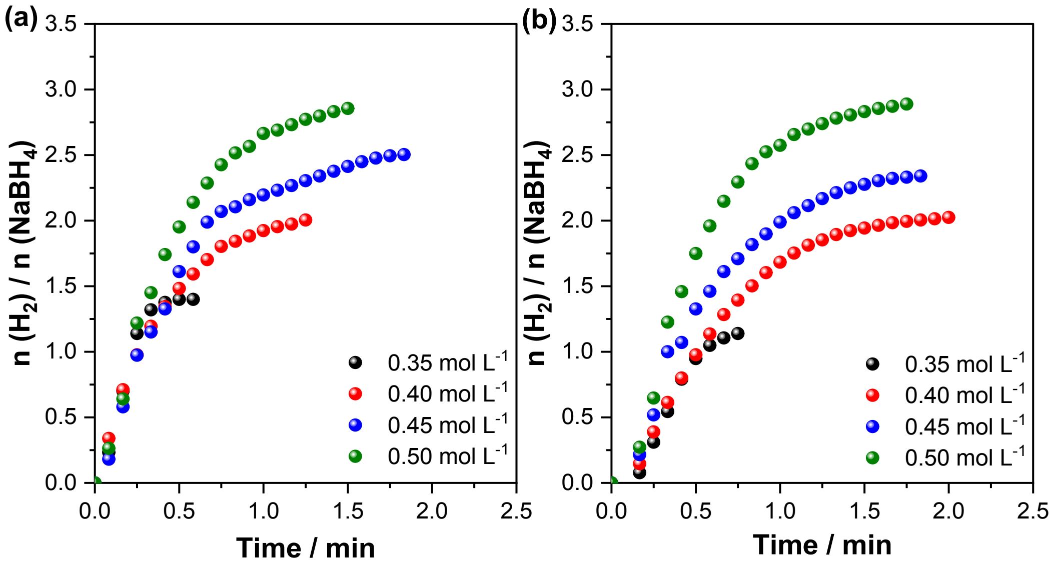

Figure S10. Hydrogen evolution from different $NaBH_4$ concentration by metallic nanoparticles decorated on (a) $NaNbO_3$ and (b) $LiNbO_3$. Experimental conditions: 5 mg of support, 0.1067 mmol of catalyst, 1.0 mL $NaBH_4$ (0.500 mol L^{-1}), constant agitation, and temperature of 298.15 K.

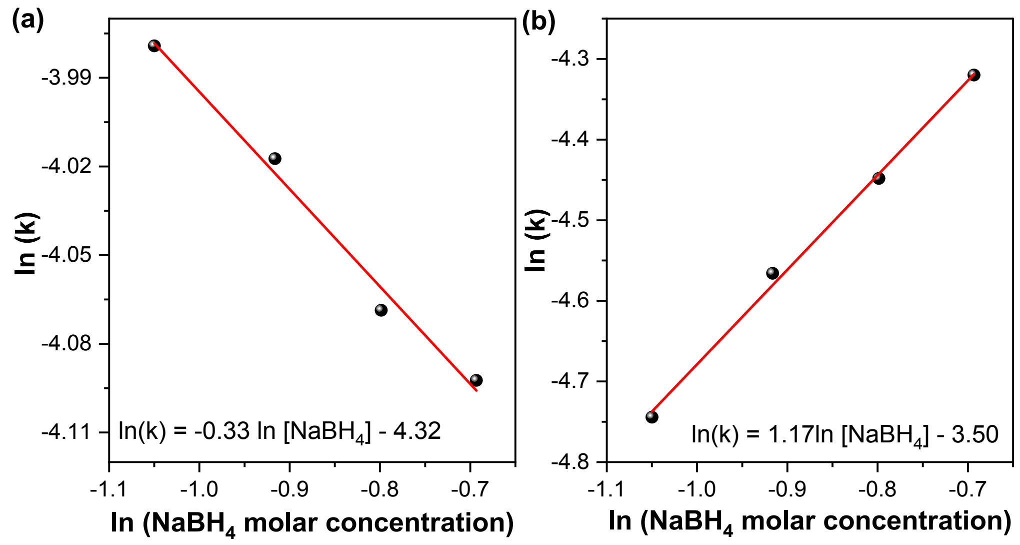

Figure S11. ln (k) *vs* ln ($NaBH_4$ molar concentration) of (a) Pt NPs/$NaNbO_3$ and (b) Pt NPs/$LiNbO_3$. Experimental conditions: 5 mg of support, 0.1067 mmol of Pt, 1.0 mL $NaBH_4$ (0.500 mol L^{-1}), constant agitation, and temperature of 298.15 K.

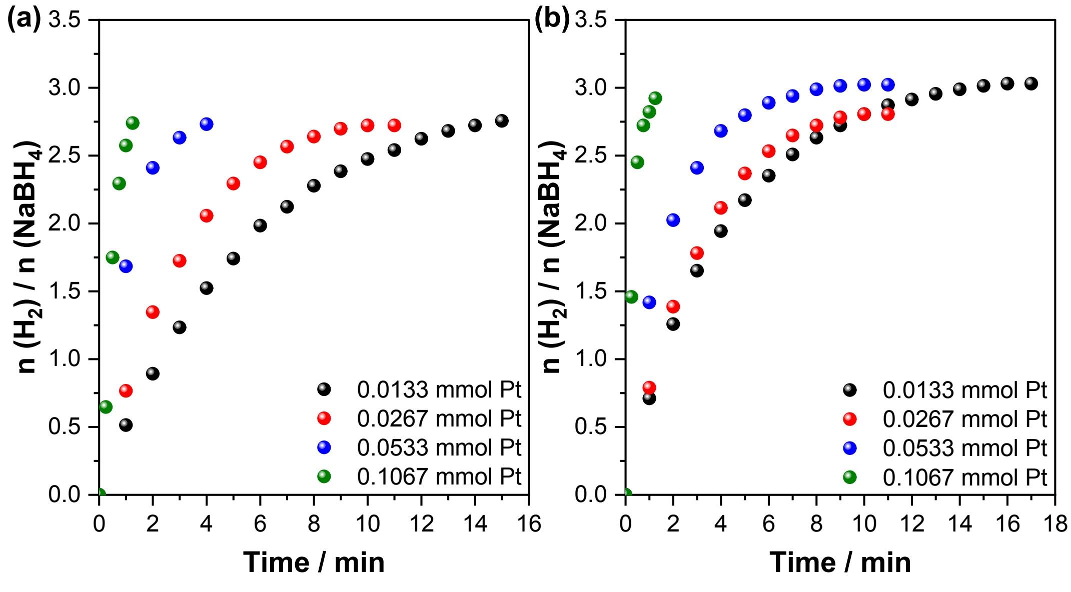

Figure S12. Hydrogen evolution from the hydrolysis of $NaBH_4$ at different catalyst dosages (0.0133, 0.0267, 0.0533 and 0.1067 mmol Pt). (a) Pt NPs/$NaNbO_3$ and (b) Pt NPs/$LiNbO_3$. Experimental conditions: 5 mg of support, 1.0 mL $NaBH_4$ (0.500 mol L^{-1}), constant agitation, and temperature of 298.15 K.

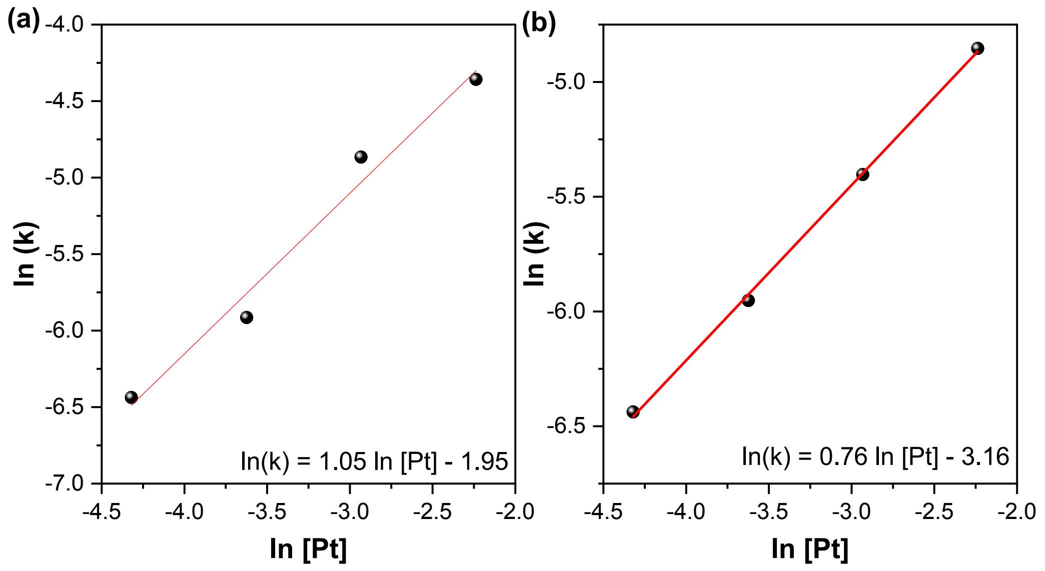

Figure S13. ln (k) *vs* ln (catalyst dosage) of (a) Pt NPs/$NaNbO_3$ and (b) Pt NPs/$LiNbO_3$. Experimental conditions: 5 mg of support, 0.1067 mmol of Pt, 1.0 mL $NaBH_4$ (0.500 mol L^{-1}), constant agitation, and temperature of 298.15 K.

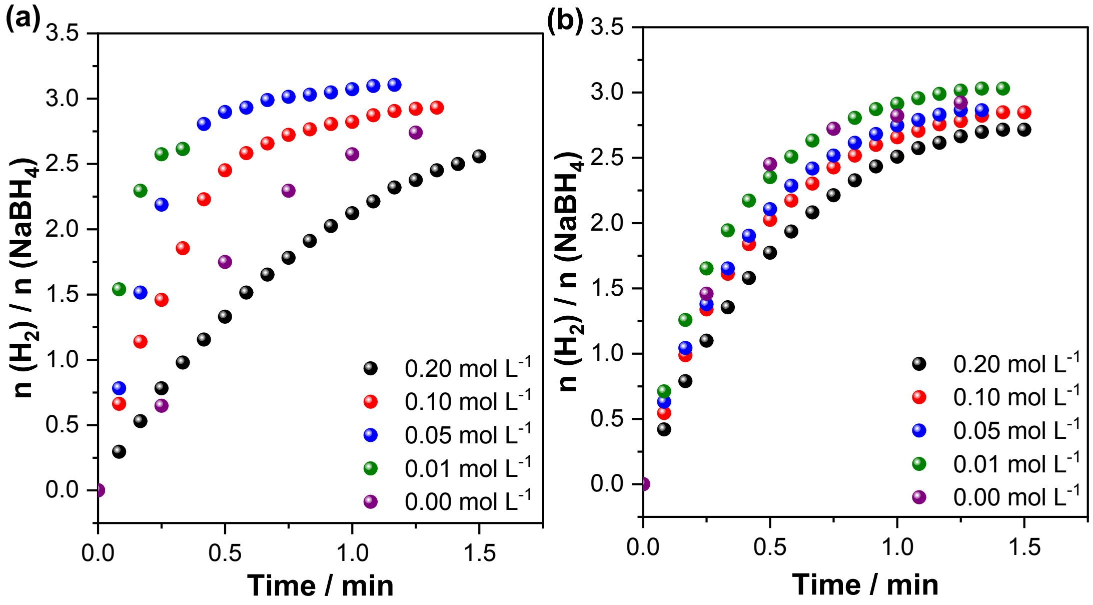

Figure S14. Hydrogen evolution from the hydrolysis of $NaBH_4$ at different NaOH concentration using (a) Pt NPs/$NaNbO_3$ and (b) Pt NPs/$LiNbO_3$. Experimental conditions: 5 mg of support, 0.1067 mmol of catalyst, 1.0 mL $NaBH_4$ (0.500 mol L^{-1}), constant agitation, and temperature of 298.15 K.

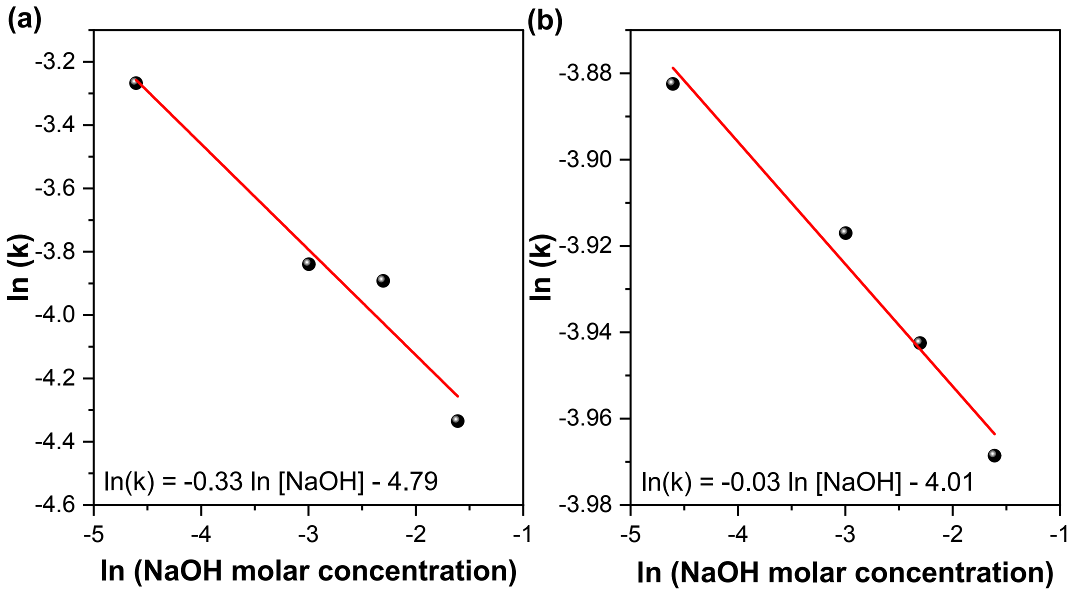

Figure S15. ln (k) *vs* ln (NaOH molar concentration) of (a) Pt NPs/$NaNbO_3$ and (b) Pt NPs/$LiNbO_3$. Experimental conditions: 5 mg of support, 0.1067 mmol of Pt, 1.0 mL $NaBH_4$ (0.500 mol L^{-1}) in NaOH solutions, constant agitation, and temperature of 298.15 K.

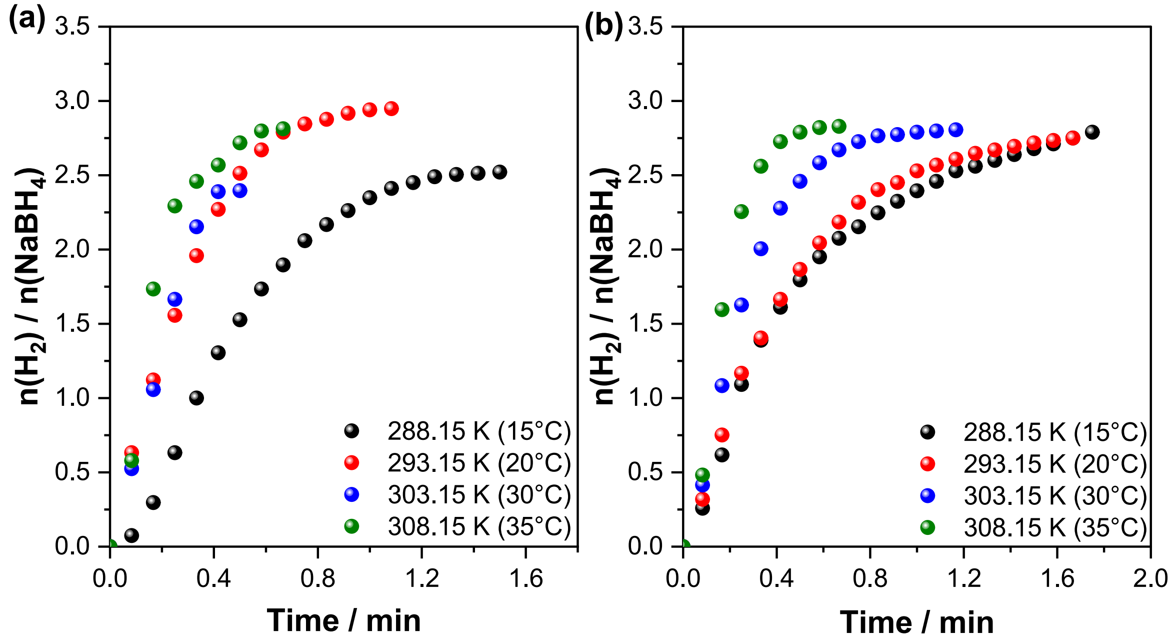

Figure S16. Evaluation of temperature in hydrogen evolution from $NaBH_4$ by (a) Pt NPs/$NaNbO_3$ and (b) Pt NPs/$LiNbO_3$. Experimental conditions: 5 mg of support mass, 0.1067 mmol of Pt, 1.0 mL $NaBH_4$ (0.500 mol L^{-1}), constant agitation.

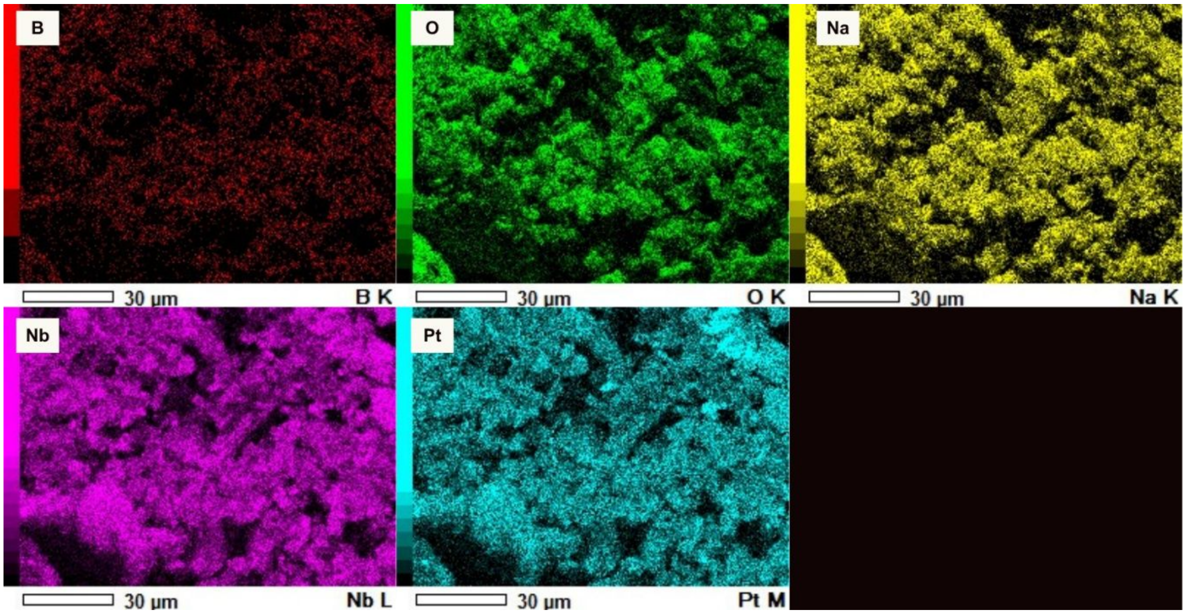

Figure S17. Elemental mapping of Pt NPs-$NaNbO_3$ after the catalyst reusability obtained through energy-dispersive X-ray spectroscopy (EDS) analysis.

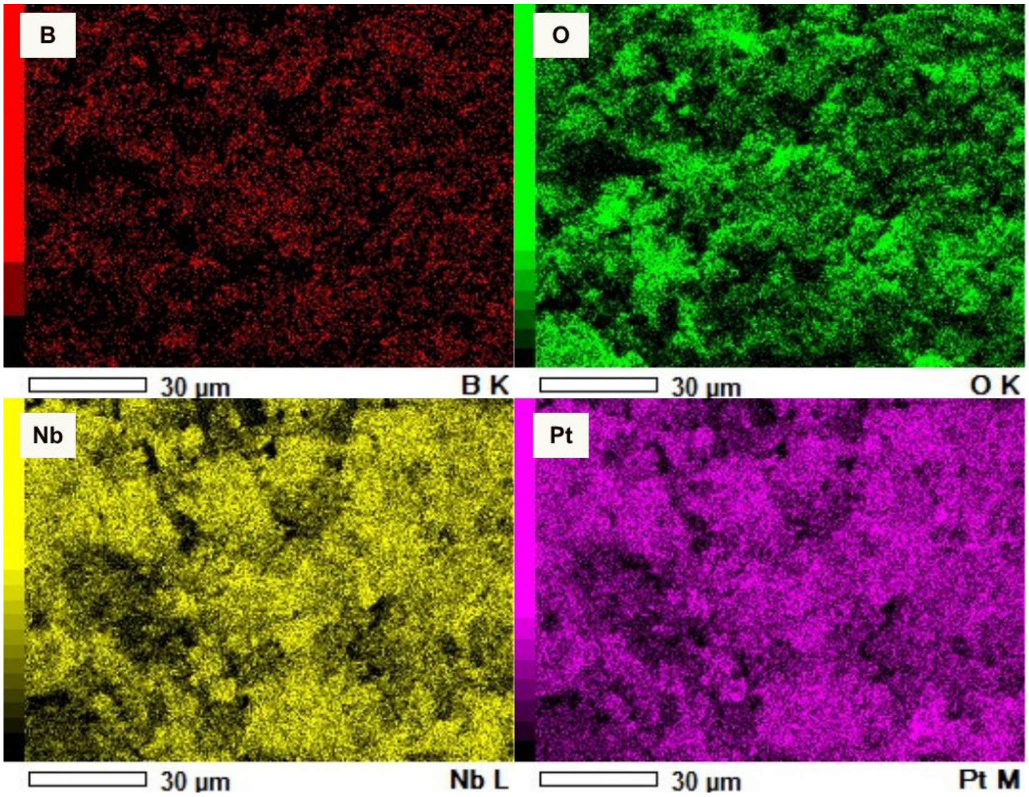

Figure S18. Elemental mapping of Pt NPs-$LiNbO_3$ after the catalyst reusability obtained through energy-dispersive X-ray spectroscopy (EDS) analysis.

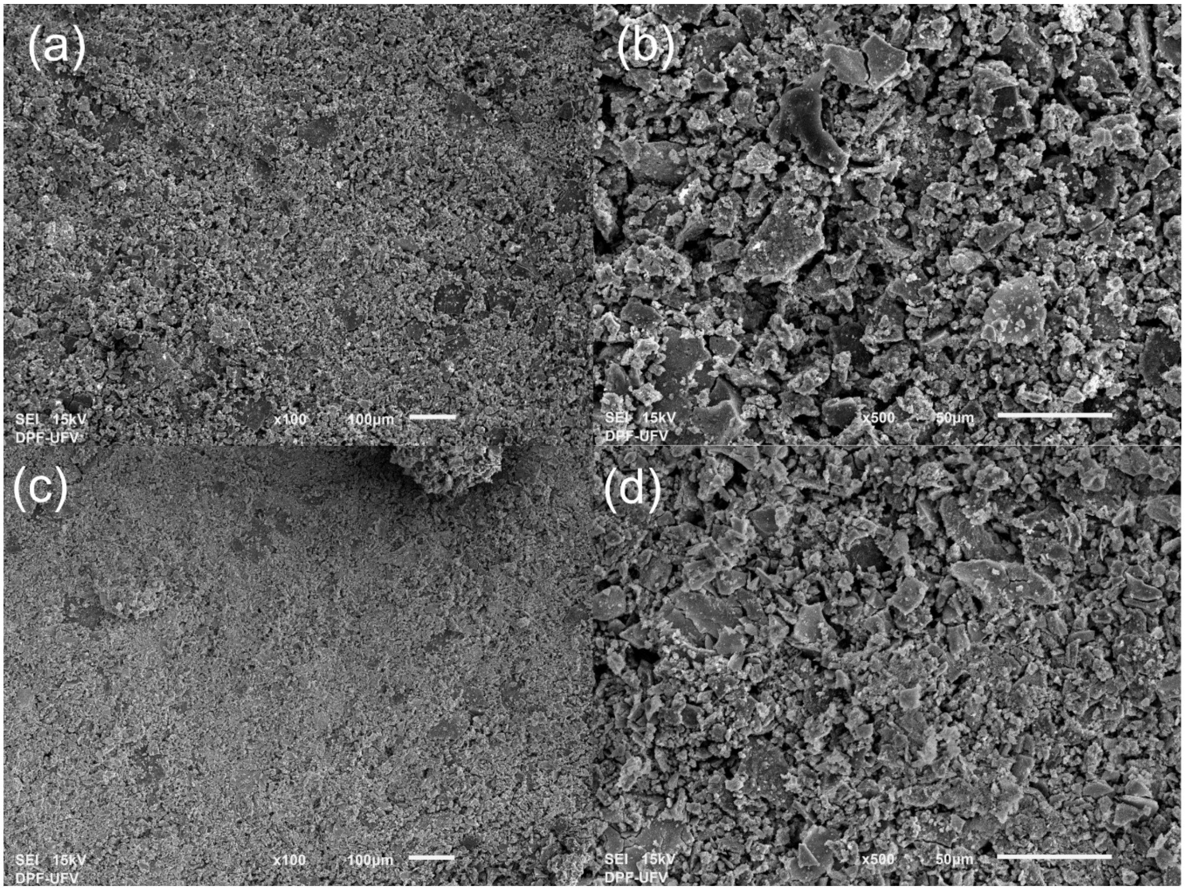

Figure S19. Scanning Electron Microscopy (SEM) images: (a) and (b) Pt NPs-$NaNbO_3$ after the catalyst reusability; (c) and (d) Pt NPs-$LiNbO_3$ after the catalyst reuse, with 100× and 500× magnifications, respectively.
